# The conserved Mediator subunit MDT-15 is required for oxidative stress responses in *Caenorhabditis elegans*

**DOI:** 10.1111/acel.12154

**Published:** 2013-09-18

**Authors:** Grace Y S Goh, Katherine L Martelli, Kulveer S Parhar, Ada W L Kwong, Marcus A Wong, Allan Mah, Nicole S Hou, Stefan Taubert

**Affiliations:** 1 Graduate Program in Cell and Developmental Biology, University of British ColumbiaVancouver, BC, Canada; 2 Centre for Molecular Medicine and Therapeutics, Child & Family Research InstituteVancouver, BC, Canada; 3 Department of Medical Genetics, University of British ColumbiaVancouver, BC, Canada

**Keywords:** *daf-2*, MDT-15, Mediator complex, nuclear hormone receptor, oxidative stress, SKN-1

## Abstract

Reactive oxygen species (ROS) play important signaling roles in metazoans, but also cause significant molecular damage. Animals tightly control ROS levels using sophisticated defense mechanisms, yet the transcriptional pathways that induce ROS defense remain incompletely understood. In the nematode *Caenorhabditis elegans*, the transcription factor SKN-1 is considered a master regulator for detoxification and oxidative stress responses. Here, we show that MDT-15, a subunit of the conserved Mediator complex, is also required for oxidative stress responses in nematodes. Specifically, *mdt-15* is required to express SKN-1 targets upon chemical and genetic increase in SKN-1 activity. *mdt-15* is also required to express genes in SKN-1-dependent and SKN-1-independent fashions downstream of insulin/IGF-1 signaling and for the longevity of *daf-2*/insulin receptor mutants. At the molecular level, MDT-15 binds SKN-1 through a region distinct from the classical transcription-factor-binding KIX-domain. Moreover, *mdt-15* is essential for the transcriptional response to and survival on the organic peroxide tert-butyl-hydroperoxide (tBOOH), a largely SKN-1-independent response. The MDT-15 interacting nuclear hormone receptor, NHR-64, is specifically required for tBOOH but not arsenite resistance, but NHR-64 is dispensable for the transcriptional response to tBOOH. Hence, NHR-64 and MDT-15’s mode of action remain elusive. Lastly, the role of MDT-15 in oxidative stress defense is functionally separable from its function in fatty acid metabolism, as exogenous polyunsaturated fatty acid complementation rescues developmental, but not stress sensitivity phenotypes of *mdt-15* worms. Our findings reveal novel conserved players in the oxidative stress response and suggest a broad cytoprotective role for MDT-15.

## Introduction

Reactive oxygen species (ROS) are ubiquitous molecules that occur as byproducts of aerobic metabolism. ROS have important biological properties and activities: on one hand, they serve as signaling molecules in regulatory circuits; on the other hand, they can damage cellular macromolecules due to their reactive nature (Hekimi *et al*., 2011; Back *et al*., 2012). Aberrant ROS accumulation causes oxidative stress, which is implicated in the development or aggravation of cancer, diabetes, and neurodegenerative diseases (Zhang *et al*., 2011). To protect against oxidative stress, eukaryotes possess sophisticated defense systems that cope with elevated ROS levels and promote homeostasis. Proteins that protect against high ROS levels include catalases, superoxide dismutases (SODs), and glutathione S-transferases (GSTs) (Xu *et al*., 2005; Lindblom & Dodd, 2006). Many such cytoprotective genes are transcriptionally induced by oxidative stress, and numerous transcription factors are required to activate overlapping, but distinct gene sets upon oxidative stress (Ma, 2010). Defining the transcriptional regulatory networks that limit cellular damage is thus an important scientific question.

The genetically tractable nematode *Caenorhabditis elegans* provides an excellent model to dissect cytoprotective circuits *in vivo*. In *C. elegans*, the transcriptional response to oxidative stress is mediated by conserved transcription factors such as the basic-leucine zipper type protein SKN-1 and the Forkhead BoxO (FoxO) type protein DAF-16 (An & Blackwell, 2003; Murphy *et al*., 2003; Ma, 2010). Both factors control the expression of detoxification enzymes and are required for resistance to heat, oxidative, and other stresses, as well as for the expression of cytoprotective genes in long-lived worm mutants (An & Blackwell, 2003; Murphy *et al*., 2003; Tullet *et al*., 2008; Oliveira *et al*., 2009). However, the regulatory complexes that specify the transcriptional responses to individual oxidative stressors remain poorly defined, although different types of oxidative stress activate distinct gene expression signatures (Oliveira *et al*., 2009; Park *et al*., 2009; Przybysz *et al*., 2009).

The multiprotein Mediator complex is an evolutionarily conserved transcriptional regulatory complex that is important for basal and activated transcription and is targeted by many transcription factors (Malik & Roeder, 2010; Conaway & Conaway, 2011). Although the complex as a whole is universally required for transcription, some Mediator subunits act in a selective fashion (Malik & Roeder, 2010; Conaway & Conaway, 2011). In *C. elegans*, the Mediator subunit MDT-15 is specifically required to express fatty acid metabolism genes, as well as fasting response, heavy metal detoxification, and xenobiotic detoxification genes (Taubert *et al*., 2006, 2011; Yang *et al*., 2006). MDT-15 cooperates with SBP-1, the *C. elegans* orthologue of sterol regulatory element binding proteins (SREBPs) and with the nuclear hormone receptor NHR-49 to regulate fatty acid metabolism genes. However, neither SBP-1, NHR-49, nor the detoxification regulator NHR-8 (Lindblom & Dodd, 2006) affect MDT-15-dependent detoxification genes (Taubert *et al*., 2008), suggesting that MDT-15 must interact with yet unidentified transcription factors to regulate stress response genes.

The biological functions of MDT-15 are evolutionarily conserved, as its yeast orthologue Gal11 is also required to express genes involved in lipid and drug metabolism and stress resistance (Thorpe *et al*., 2004; Thakur *et al*., 2008, 2009). To regulate lipid and drug metabolism genes, Gal11 cooperates with the zinc cluster transcription factors Pdr1 and Oaf1 (Thakur *et al*., 2008, 2009). Zinc cluster proteins are distantly related to metazoan nuclear hormone receptors (Naar & Thakur, 2009), suggesting that MDT-15 and its metazoan counterparts may regulate similar responses by involving such proteins.

Many detoxification genes are required for the response to oxidative stress, and we previously showed that *mdt-15* is required to express many such genes (Taubert *et al*., 2008). Thus, we set out to test whether *mdt-15* is required for oxidative stress response programs in *C. elegans* and to identify transcription factors that cooperate with MDT-15 to selectively induce stress responses.

## Results

### *mdt-15* is required for survival in oxidative stress

To test whether MDT-15 is involved in oxidative stress responses, we compared MDT-15-dependent genes and oxidative stress response genes. Specifically, we looked for overlaps between genes downregulated following *mdt-15* depletion and genes upregulated by exposure to hyperoxia, arsenite, tert-butyl hydroperoxide (tBOOH), or juglone. We found a statistically significant overlap in three of four cases (Table 1 and Table S1).

**Table 1:** Overlap between MDT-15-dependent genes and genes induced by oxidative stress, and between MDT-15- and SKN-1-dependent genes

	Down in *mdt-15* RNAi (187 genes, Taubert *et al*., 2008)
Arsenite induced (118 genes, Oliveira *et al*., 2009)	Expected overlap: 2.0
	*Actual overlap: 11 (10 have predicted SKN-1-binding sites)*
	*P*-value 5.44 e-06
tBOOH induced (285 genes, Oliveira *et al*., 2009)	Expected overlap: 4.9
	*Actual overlap: 9*
	*P*-value 0.057
Hyperoxia (100% O_2_) induced (948 genes, Park *et al*., 2009)	Expected overlap: 16.3
	*Actual overlap: 37*
	*P*-value 2.17E-06
Juglone induced in L4 (103 genes, Przybysz *et al*., 2009)	Expected overlap: 1.8
	*Actual overlap: 10*
	*P*-value 1.02E-05
SKN-1 induced (233 genes, Oliveira *et al*., 2009)	Expected overlap: 4.0
	*Actual overlap: 13 (10 have predicted SKN-1-binding sites)*
	*P*-value 1.85E-04

The table shows the number of genes expected to overlap between lists of relevant sizes, the number of genes that actually overlap, and *P*-values (Fisher’s exact test) indicating significance of said overlap. The expected overlap is the fraction of MDT-15-regulated genes multiplied with the fraction of stress responsive genes.

**Figure 1:**
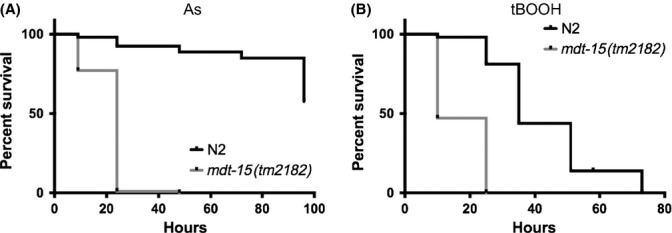
*mdt-15* worms are sensitive to oxidative stress. Survival plots of wild-type N2 and *mdt-15(rf)* worms on (A) 5 mm arsenite and (B) 6 mm tBOOH. Tables S2 and S3 show statistics and replicates.

To test whether these gene expression changes cause oxidative stress sensitivity, we quantified population survival of wild-type worms and *mdt-15(tm2182)* mutants (Taubert *et al*., 2008; henceforth, *mdt-15(rf)* mutants, see below) on inorganic arsenite and on the organic peroxide tBOOH. We found that *mdt-15(rf)* mutants were hypersensitive to both stressors (Fig. 1, Tables S2 and S3). Depleting *mdt-15* by RNA interference (RNAi) also caused tBOOH sensitivity (Fig. 4A). Thus, *mdt-15* is required for normal oxidative stress resistance.

### MDT-15 is essential for the transcriptional response to arsenite

The oxidative stress sensitivity of *mdt-15(rf)* mutants could be due to reduced expression of stress regulators such as SKN-1 or DAF-16 (An & Blackwell, 2003; Murphy *et al*., 2003), changes in fatty acid metabolism (Taubert *et al*., 2006; Yang *et al*., 2006), or a requirement for MDT-15 in the induction of stress response genes. To test whether MDT-15 affects *skn-1* and *daf-16* expressions, we used real-time PCR (qPCR) to quantify their mRNA levels *in vivo*. *mdt-15* depletion or mutation did not significantly alter *skn-1* levels and actually increased *daf-16* levels (Fig. S1A,B). The levels and the nuclear localization of DAF-16::GFP (Henderson & Johnson, 2005) and SKN-1::GFP (An & Blackwell, 2003) were also similar in *control(RNAi)* and *mdt-15(RNAi)* worms (Fig. S1C,D). Thus, the phenotypes of *mdt-15(RNAi)* and *mdt-15(rf)* worms are unlikely to originate from compromised SKN-1 or DAF-16 expression or localization.

**Figure 2:**
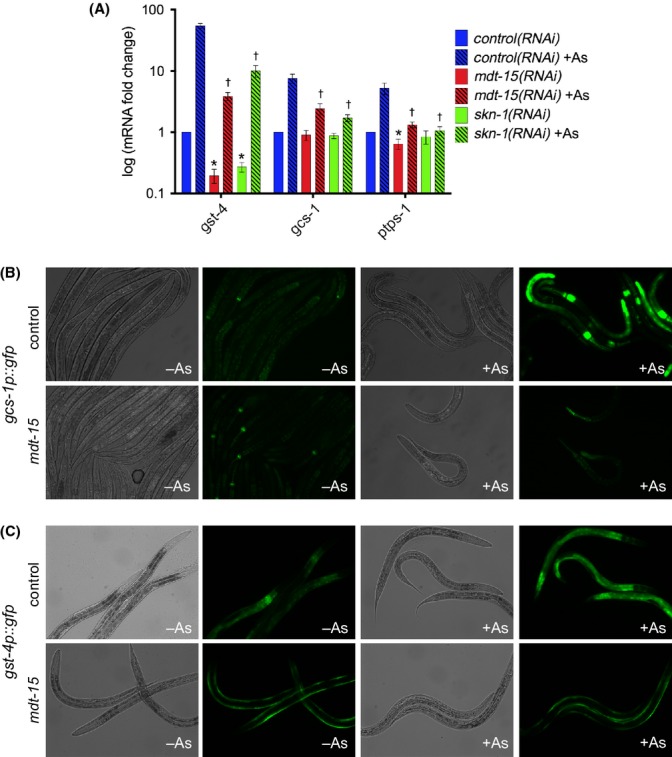
*mdt-15* is required for the transcriptional response to arsenite. (A) Fold changes of mRNA levels (relative to untreated *control(RNAi)*) in L4 wild-type worms grown on control, *mdt-15,* or *skn-1* RNAi and treated with 5 mm sodium arsenite for 4 h (*n* = 5). mRNA levels were normalized to *act-1*, *ama-1*, *cdc-42*, and *tba-1*; error bars represent SEM. *Untreated *mdt-15(RNAi)* or *skn-1(RNAi)* worms differ significantly from *control(RNAi)* worms (*P* < 0.05). ^†^Treated *mdt-15(RNAi)* or *skn-1(RNAi)* worms differ significantly from *control(RNAi)* worms (*P* < 0.05). (B) DIC and fluorescence micrographs show *gcs-1p::gfp* worms grown on control or *mdt-15* RNAi after 4 h on 5 mm sodium arsenite. One of three repeats is shown. (C) Same as (B), except with *gst-4p::gfp* worms.

To test whether *mdt-15* is required to induce oxidative stress response genes, we grew synchronized wild-type worms to the L4 stage on control and *mdt-15* RNAi, and then exposed them to 5 mm arsenite for 4 h and used qPCR to quantify oxidative stress gene expression. Arsenite reproducibly induced six genes more than twofold in *control(RNAi)* worms, and fold inductions (not only basal levels) of four genes were significantly reduced in *mdt-15(RNAi)* worms (Fig. 2A, Fig. S2A). To support the RNAi studies, we also exposed synchronized wild-type and *mdt-15(rf)* L4 larvae to arsenite. *mdt-15(rf)* mutants showed significantly impaired arsenite inductions for five genes and reduced basal levels of three genes, resembling *mdt-15(RNAi)* worms (Fig. S2B). Importantly, we obtained similar results with a 1-h arsenite exposure (Fig. S2C), suggesting that the compromised gene induction could cause the oxidative stress sensitivity of *mdt-15(rf)* worms.

To corroborate the qPCR data, we studied worms expressing transcriptional *gcs-1p::gfp* or *gst-4p::gfp* reporters (Wang *et al*., 2010). In L4 stage *gcs-1p::gfp* worms, the induction of intestinal GFP by a 4-h arsenite exposure was severely compromised when worms were grown on *mdt-15* RNAi (Fig. 2B). To ensure that this phenotype was not caused by impaired development due to *mdt-15* depletion, we exposed late L4 *gcs-1p::gfp* worms to RNAi for 48 h and then exposed them to arsenite for 4 h. Induction of *gcs-1p::gfp* by arsenite remained *mdt-15* dependent in this adult-only RNAi regimen (Fig. S2D). We observed a similar *mdt-15* requirement in worms expressing a *gst-4p::gfp* reporter (Fig. 2C). Notably, *mdt-15* depletion reduced intestinal *gst-4p*-driven fluorescence but evoked hypodermal fluorescence not seen in *control(RNAi)* worms, perhaps reflecting compensatory, *mdt-15*-independent *gst-4* induction (Fig. 2C). Together, these data suggest that MDT-15 coregulates the transcriptional stress response to arsenite by affecting a subset of arsenite-responsive genes.

### MDT-15 is required to induce SKN-1 targets in worms with elevated SKN-1 levels

Mediator subunits are tethered to genomic regulatory elements by transcription factors. In *C. elegans*, SKN-1 is a key regulator of the arsenite response (Oliveira *et al*., 2009). To test whether SKN-1 and MDT-15 cooperate, we compared SKN-1 and MDT-15-dependent genes and found that there is a statistically significant overlap (Table 1); predicted SKN-1-binding sites (Oliveira *et al*., 2009) occur in 10 of 13 shared targets (77%), similar to the approximately 80% found in ‘SKN-1-only’ targets. Moreover, qPCR revealed that several genes respond similarly to *mdt-15* and *skn-1* depletion (Fig. 2A), and MDT-15 primarily affects intestinal *gcs-1* and *gst-4* expressions (Fig. 2B,C), like SKN-1 (An & Blackwell, 2003). This suggests that MDT-15 and SKN-1 directly coregulate some arsenite-responsive genes.

**Figure 3:**
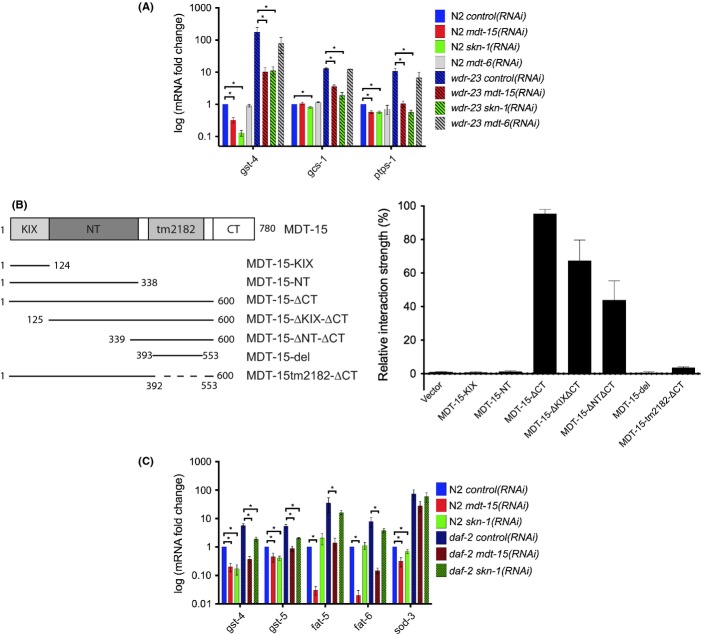
MDT-15 interacts functionally and physically with SKN-1. (A) Relative mRNA fold changes of SKN-1 targets in L4 stage wild-type N2 or *wdr-23(−)* worms grown on either control, *mdt-15*, *skn-1*, or *mdt-6* RNAi (*n* = 4). mRNA levels were normalized to *act-1*, *ama-1*, *cdc-42*, and *tba-1*; error bars represent SEM. * indicates *P* < 0.05. (B) Relative interaction strength between Gal4DBD-MDT-15 variants and Gal4AD-SKN-1c; the schematic depicts MDT-15 fusion proteins. Values indicate average interaction strength in percent, calculated from Miller units (*n* > 4 per plasmid combination); error bars represent SEM. Protein expression of Gal4DBD-MDT-15 fusions was confirmed by immunoblot (Fig. S4). (C) Relative mRNA fold changes in L2 stage N2 and *daf-2(e1370)* worms grown on control, *mdt-15*, or *skn-1* RNAi (*n* = 4). mRNA levels were normalized to *act-1*, *ama-1*, *cdc-42,* and *tba-1*; error bars represent SEM. * indicates *P* < 0.05.

To test whether MDT-15 is a SKN-1 coregulator, we studied *wdr-23(tm1817)* loss-of-function mutants (Choe *et al*., 2009). WDR-23 is part of an ubiquitin ligase complex that promotes SKN-1 degradation; thus, *wdr-23(−)* worms exhibit increased levels of SKN-1 and SKN-1 target genes. If *mdt-15* were required to express SKN-1 targets, *mdt-15* depletion should suppress SKN-1-dependent gene inductions in *wdr-23(−)* mutants. To test this hypothesis, we quantified mRNA levels in developmentally synchronized wild-type and *wdr-23(−)* worms grown on control, *mdt-15*, and *skn-1* RNAi. We found that the inductions of five SKN-1 targets in *wdr-23(−)* mutants were strongly and similarly reduced by *mdt-15* and *skn-1* depletion (Fig. 3A, Fig. S3A). We also depleted *wdr-23* in *mdt-15(rf)* mutants and found that *mdt-15(rf)* mutants exhibit significantly impaired induction of SKN-1 targets on *wdr-23* RNAi (Fig. S3B). Thus, increased expression of SKN-1 targets in *wdr-23* worms requires *mdt-15*.

Some Mediator subunits act in gene-specific fashion, but others are broadly required for transcription. To address subunit specificity in SKN-1 target gene transcription, we tested whether MDT-6 was required for the SKN-1-dependent inductions in *wdr-23(−)* mutants. Unlike *mdt-15*, *mdt-6* RNAi did not prevent the induction of SKN-1 targets in *wdr-23* mutants (Fig. 3A), although both RNAi clones delay growth and development. These data provide evidence for Mediator subunit specificity and demonstrate that developmental arrest *per se* is not sufficient to block SKN-1 target induction.

### MDT-15 physically associates with SKN-1 via non-KIX-domain interactions

To test whether SKN-1 physically binds MDT-15, we used the yeast-two-hybrid system. MDT-15 contains an N-terminal KIX-domain that binds nuclear hormone receptors (NHRs) and the lipogenic transcription factor SBP-1 (Taubert *et al*., 2006; Yang *et al*., 2006). As this is the only recognized transcription-factor-binding domain in MDT-15, we hypothesized that the KIX-domain (aa1-124; Fig. 3B) might associate physically with SKN-1. However, an MDT-15-KIX bait did not interact with SKN-1 in our yeast-two-hybrid assays (Fig. 3B, Fig. S4A).

The yeast MDT-15 orthologue Gal11 uses multiple surfaces to bind the transcription factor Gcn4, with the KIX-domain playing a minor role (Herbig *et al*., 2010; Jedidi *et al*., 2010). Thus, we tested whether SKN-1 interacted with two longer baits, MDT-15-NT (aa 1-338), and near-full-length MDT-15-ΔCT (aa 1-600; Fig. 3B; full-length MDT-15 autoactivates and cannot be used as bait). While MDT-15-NT failed to interact, MDT-15ΔCT strongly and specifically bound SKN-1c (Fig. 3B; SKN-1a and SKN-1b were undetectable).

The KIX-domain is not sufficient for SKN-1 binding, but might be required. To test this hypothesis, we assayed binding of SKN-1c to an MDT-15ΔCT variant lacking the KIX-domain (MDT-15ΔKIXΔCT; aa 125-600). Binding of SKN-1c to MDT-15ΔKIXΔCT was as strong as binding to MDT-15ΔCT (Fig. 3B), indicating that the KIX-domain is dispensable for SKN-1c binding.

### The region deleted in *mdt-15* mutants is required for SKN-1 binding

As *mdt-15(rf)* mutants fail to activate some SKN-1 targets, we hypothesized that the MDT-15 protein they produce might be unable to bind SKN-1. Immunoblot analysis with antibodies against the MDT-15 N-terminus (Taubert *et al*., 2006) revealed that *mdt-15(rf)* worms produce an approximately 70 kDa protein instead of approximately 85 kDa wild-type MDT-15 (Fig. S5A). Sequencing the *mdt-15* cDNA from *mdt-15(rf)* mutants (*mdt-15* RNA and proteins are expressed at comparable levels in WT and *mdt-15(rf)* worms; Fig. S5A,B) revealed that the *tm2182* allele is an in-frame deletion and insertion that produces a 618 aa protein with a predicted molecular weight of 66 kDa (Fig. S5C), reflecting the band detected by immunoblot. To study MDT-15tm2182 function, we asked whether a Gal4DBD-MDT-15tm2182 fusion protein activated a Gal4-driven β-galactosidase reporter in yeast. Gal4DBD-MDT-15tm2182 activated as well as Gal4DBD-MDT-15 (Fig. S5D), suggesting it is not generally dysfunctional. In sum, MDT-15tm2182 is expressed at wild-type levels *in vivo* and is transcriptionally competent. Along with the fact that the phenotypes of *mdt-15(rf)* worms are weaker than those of *mdt-15(RNAi)* worms (Taubert *et al*., 2008), this suggests that *tm2182* is a hypomorph, not a loss-of-function allele.

Next, we addressed whether the region deleted in *mdt-15(rf)* worms is involved in SKN-1 binding. We found that an MDT-15tm2182ΔCT bait (aa 1-392–553-600) recapitulating the *tm2182* deletion showed weak SKN-1c binding compared with MDT-15ΔCT (Fig. 3B). Thus, the *tm2182* mutation compromises MDT-15 binding to SKN-1c, providing a molecular explanation for the inability of *mdt-15(rf)* worms to induce SKN-1 targets. We also tested whether the deleted region (MDT-15-del; aa 393-552) was sufficient for SKN-1c interaction, but it failed bind SKN-1c above background levels, suggesting that it is not (Fig. 3B).

### MDT-15 regulates gene expression downstream of DAF-2

Besides activating stress response genes, *skn-1* is required for gene inductions and longevity in worms carrying a mutation in the insulin/IGF-1 receptor gene *daf-2* (Kenyon *et al*., 1993; Tullet *et al*., 2008). To test whether MDT-15 coregulates SKN-1 in this context, we quantified gene expression in wild-type and *daf-2(e1370)* worms grown on control, *skn-1*, and *mdt-15* RNAi by qPCR. Tullet *et al*. found several *gst* genes to be upregulated in *daf-2(e1370)* mutants in a *skn-1*-dependent fashion; we found that *mdt-15* depletion also significantly reduced these inductions (Fig. 3C, Fig. S6A). *mdt-15* depletion additionally affected SKN-1-independent genes such as *fat-5* and *fat-6* (Taubert *et al*., 2006; Fig. 3C, Fig. S6A). Thus, MDT-15 regulates stress response and lipid metabolism genes in SKN-1-dependent and SKN-1-independent fashion in *daf-2* mutants.

The transcription factor DAF-16 modulates gene expression downstream of *daf-2* and hence might cooperate with MDT-15 to regulate SKN-1-independent genes. We found that mRNA induction of the direct DAF-16 target *sod-3* was slightly reduced in *daf-2(e1370)* mutants grown on *mdt-15* RNAi (Fig. 3C). Similarly, *daf-2(e1370)* worms expressing a transgenic *sod-3p::gfp* reporter (Libina *et al*., 2003) showed reduced intestinal GFP fluorescence on *mdt-15* RNAi (Fig. S6B). However, DAF-16 did not bind MDT-15 in our yeast-two-hybrid assays (data not shown), and the effect of MDT-15 depletion on *sod-3* mRNA levels was relatively weak, suggesting that MDT-15 only plays a minor role in DAF-16-dependent transcription.

As MDT-15 is required for gene expression in *daf-2* mutants (Fig. 3C), we examined two phenotypes linked to *daf-2*, longevity and dauer larvae formation (Fielenbach & Antebi, 2008). As published by Zhang *et al*. (2013), adult-only *mdt-15* RNAi reduced the lifespan of *daf-2(e1370)* and *daf-2(e1368)* mutants, but also reduced wild-type lifespan (Fig. S6B,C). To test whether *mdt-15* is required for dauer formation, we exposed wild-type and *mdt-15(rf)* worms to dauer-inducing ascaroside #2 (Butcher *et al*., 2007), and found that they entered and exited dauer with wild-type kinetics and frequency (data not shown). Thus, the role of *mdt-15* in insulin signaling resembles that of *skn-1*, which is required for longevity but dispensable for dauer formation (Tullet *et al*., 2008).

### Altered fatty acid metabolism in *mdt-15* worms does not cause tBOOH sensitivity

**Figure 4:**
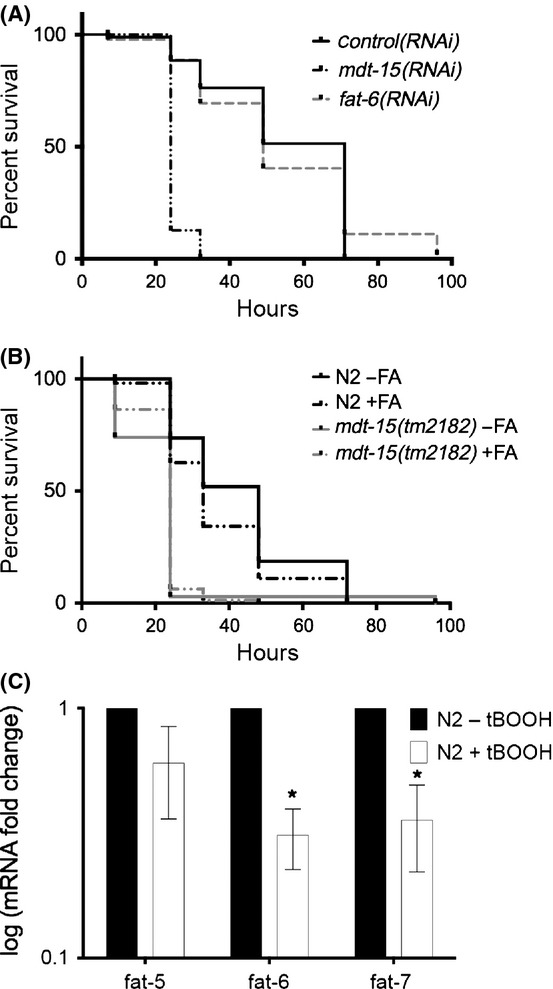
MDT-15 regulates oxidative stress responses independently of fatty acid desaturation. (A) Survival plots of *control(RNAi)*, *mdt-15(RNAi)*, and *fat-6(RNAi)* worms on 6 mm tBOOH. Table S4 shows statistics and replicates. (B) Survival plots of wild-type N2 and *mdt-15(tm2182)* worms on 6 mm tBOOH following development on PUFAs (300 μm oleic acid, 300 μm linoleic acid, and 100 μm eicosapentaenoic acid). Table S5 shows statistics and replicates. (C) Relative mRNA fold changes of *fat-5*, *-6*, and *-7* in wild-type worms in unstressed conditions and after 4 h on 7.5 mm tBOOH (*n* = 4). mRNA levels were normalized to *act-1*, *tba-1,* and *ubc-2*; error bars represent SEM. *indicates *P* < 0.05.

Our data indicate that MDT-15 and SKN-1 coregulate some arsenite response genes. However, unlike *mdt-15*, *skn-1* is largely dispensable for the tBOOH response (Oliveira *et al*., 2009), implicating SKN-1-independent mechanisms for MDT-15 in this context. One possibility is that the altered fatty acid profiles of *mdt-15* worms (Taubert *et al*., 2006; Yang *et al*., 2006) underlie their tBOOH sensitivity. The fatty acid desaturases *fat-6* and *fat-7* are MDT-15 targets (Taubert *et al*., 2006; Yang *et al*., 2006), and RNAi against either enzyme causes sensitivity against the oxidative stressor paraquat (Horikawa & Sakamoto, 2009). To test whether fatty acid desaturases are required for tBOOH resistance, we exposed young adult *fat-6(tm331); fat-7(wa36)* double mutants (Brock *et al*., 2007) to tBOOH. Surprisingly, these worms were less tBOOH sensitive than WT (Fig. S7A), perhaps because their unusual C18 polyunsaturated fatty acids (PUFAs) substitute for normal C20 PUFAs. *fat-6* RNAi also failed to evoke tBOOH sensitivity, despite delaying development (Fig. 4A, Fig. S7B). This suggests that reduced *fat-6* and *fat-7* expressions do not cause tBOOH sensitivity in *mdt-15* worms. To corroborate these data, we allowed wild-type and *mdt-15(rf)* worms to complete development in the presence of exogenous PUFAs and then exposed these rescued worms to tBOOH. Although fertility, mobility, and development were improved in *mdt-15(rf)* mutants, they remained fully tBOOH sensitive (Fig. 4B). Thus, reduced PUFA levels are unlikely to cause the tBOOH sensitivity of *mdt-15(rf)* mutants. In fact, we observed that wild-type worms were slightly tBOOH hypersensitive in the presence of exogenous PUFAs (Fig. 4B). Furthermore, *fat-5*, *fat-6*, and *fat-7* mRNA levels decreased following tBOOH exposure (Fig. 4C). Taken together, these data argue that altered fatty acid profiles are unlikely to cause the tBOOH sensitivity of *mdt-15* worms.

### MDT-15-binding NHRs are required for normal oxidative stress resistance

**Figure 5:**
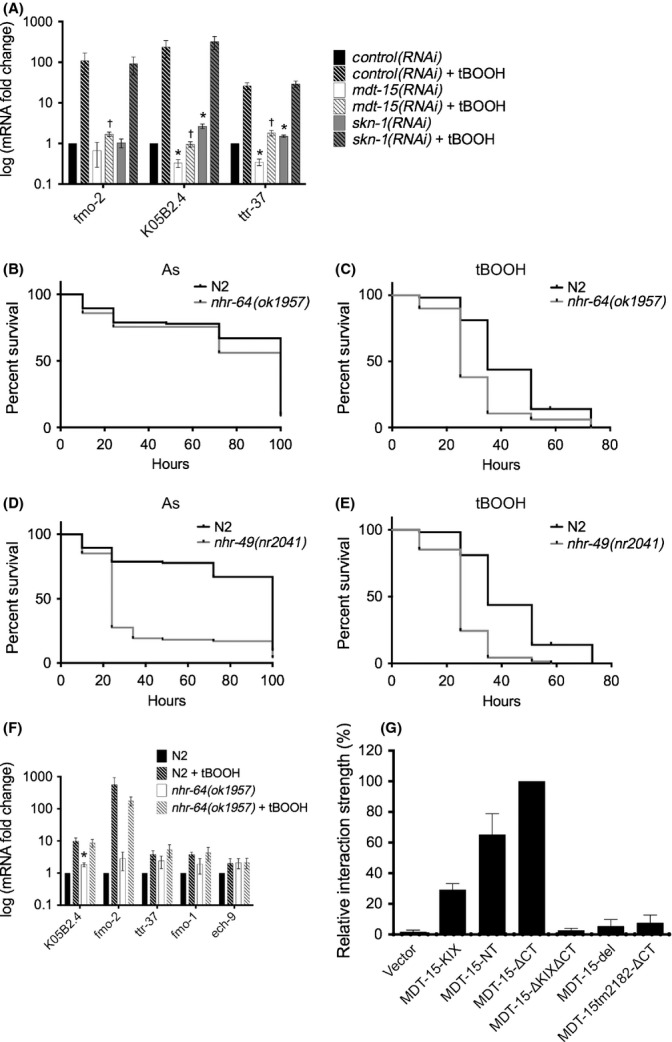
MDT-15-interacting NHRs are required for oxidative stress resistance. (A) Fold changes of mRNA levels in L4 wild-type N2 worms grown on control, *mdt-15*, or *skn-1* RNAi and treated with 7.5 mm tBOOH for 4 h, relative to untreated *control(RNAi)* worms (*n* = 4). mRNA levels were normalized to *act-1*, *ama-1*, *cdc-42,* and *tba-1*; error bars represent SEM. *Untreated *mdt-15(RNAi)* or *skn-1(RNAi)* worms differ significantly from *control(RNAi)* (*P* < 0.05). ^†^Treated *mdt-15(RNAi)* or *skn-1(RNAi)* worms differ significantly from *control(RNAi)* worms (*P* < 0.05). (B, C) Survival plots of wild-type N2 and *nhr-64(−)* worms on 5 mm arsenite and 6 mm tBOOH. (D, E) Survival plots of wild-type N2 and *nhr-49(−)* worms on 5 mm arsenite and 6 mm tBOOH. For B–E, Tables S2 and S3 show statistics and replicates. (F) mRNA fold changes in L4 stage N2 and *nhr-64(−)* worms treated with 7.5 mm tBOOH for 4 h, relative to untreated N2s (*n* = 4). mRNA levels were normalized to *act-1*, *tba-1,* and *ubc-2*; error bars represent SEM. *Untreated *nhr-64(−)* worms differ significantly from N2 worms (*P* < 0.05). (G) Relative interaction strength between Gal4DBD-MDT-15 variants and Gal4AD-NHR-64. Values indicate average interaction strength in percent, calculated from Miller units (*n* > 4 per plasmid combination); error bars represent SEM.

To test whether *mdt-15* is required for the transcriptional tBOOH response, we exposed L4 stage *control(RNAi)*, *mdt-15(RNAi),* and *skn-1(RNAi)* worms to 7.5 mm tBOOH for 4 h. *mdt-15*, but not *skn-1*, was required to induce some, but not all genes in response to tBOOH (Fig. 5A, Fig. S8A), again affecting both basal levels and fold inductions. We obtained similar data with *mdt-15(rf)* mutants, with three of six genes displaying MDT-15-dependent regulation in tBOOH (Fig. S8B,C; some genes show increased basal expression in *mdt-15(rf)* mutants). A 1-h tBOOH exposure also showed early *mdt-15* dependence for two genes (Fig. S8C; some genes were barely induced by 1 h). Thus, *mdt-15* is selectively required for the transcriptional response to two compounds evoking distinct oxidative stress signatures.

SKN-1 is largely dispensable for tBOOH-induced transcription, whereas MDT-15 is necessary (Fig. 5A). MDT-15-binding-transcription factors other than SKN-1 must therefore confer transcriptional tBOOH responses and tBOOH resistance. To test this hypothesis, we quantified the tBOOH sensitivity of previously characterized *nhr-64(ok1957)* and *nhr-49(nr2041)* null mutants (Van Gilst *et al*., 2005; Liang *et al*., 2010); both genes encode MDT-15-binding-transcription factors (Taubert *et al*., 2006). We found that *nhr-64(−)* mutants were sensitive to tBOOH but not to arsenite, whereas *nhr-49(−)* worms were sensitive to both molecules (Fig. 5B–E). *nhr-49* RNAi also causes paraquat sensitivity (Horikawa & Sakamoto, 2009), but prior microarray studies of *nhr-49(−)* mutants revealed no link to stress responses (Pathare *et al*., 2012), and *nhr-49(RNAi)* worms normally induce xenobiotic response genes upon toxin exposure (Taubert *et al*., 2008). Perhaps the broad oxidative stress sensitivity in *nhr-49* worms originates from their mitochondrial defects (Pathare *et al*., 2012).

NHR-64 regulates fat metabolism genes, but oxidative stress response genes were not studied (Liang *et al*., 2010). Thus, we quantified tBOOH responsive genes in wild-type worms and *nhr-64(−)* mutants by qPCR. Tested genes were only mildly affected in L4 stage *nhr-64(−)* mutants, and induction by tBOOH was not significantly compromised (Fig. 5F; note that basal levels were increased in *nhr-64(−)* mutants). Further work is required to define the molecular cause of tBOOH sensitivity in these mutants.

Aside from the KIX-domain, the MDT-15 surfaces involved in NHR-64 binding have not been comprehensively tested. We found that NHR-64 interacted with MDT-15-KIX, -NT, and -ΔCT, as expected, because they all contain the KIX-domain (Fig. 5G). Unlike SKN-1, NHR-64 failed to bind MDT-15-ΔKIXΔCT, demonstrating that the KIX-domain is sufficient and necessary for NHR-64 binding (Fig. 5G). Like SKN-1, NHR-64 only weakly bound to MDT-15tm2182-ΔCT, suggesting that the region deleted in *mdt-15(rf)* worms is involved in both NHR-64 and SKN-1 interactions (Fig. 5G). Thus, two separable regions in MDT-15 are required for NHR-64 binding, although the KIX-domain is sufficient for partial binding. Reduced binding of MDT-15 to NHR-64 (or other factors required for the tBOOH response) might be responsible for the tBOOH sensitivity of *mdt-15(rf)* mutants.

## Discussion

Reactive oxygen species possess both beneficial and detrimental properties, making tight control of their levels necessary. Here, we report a novel role for the *C. elegans* Mediator subunit MDT-15 in the oxidative stress response, involving a distinct functional region required for interactions with at least two transcription factors, including the well-characterized stress regulator SKN-1.

### *Caenorhabditis elegans* MDT-15 is required for at least two distinct oxidative stress responses

Our data show that *mdt-15* is required for two oxidative stress responses. Specifically, its mutation or depletion prevents normal gene inductions by, and renders worms sensitive to arsenite and tBOOH. The actions of MDT-15 are specific and not a consequence of sickness or impaired development because: (i) *mdt-15* RNAi in fully developed adults causes a defective arsenite response (Fig. S2D); (ii) PUFA complementation rescues the development, fertility, and mobility of *mdt-15* worms (Taubert *et al*., 2006; Yang *et al*., 2006), but does not rescue tBOOH sensitivity (Fig. 4B); (iii) *fat-6(RNAi)* worms and *fat-6; fat-7* double mutants are not susceptible to tBOOH, despite pleiotropic phenotypes resembling *mdt-15* worms (Fig. 4A, Fig. S7A); (iv) *mdt-15* depletion or mutation impairs the transcriptional response and causes sensitivity to oxidative stress (Figs 2 and 5) but does not block the transcriptional heat-shock response or affect thermotolerance (Taubert *et al*., 2008); and (v) *mdt-6* RNAi causes larval arrest yet fails to block SKN-1-dependent gene inductions in *wdr-23(−)* worms, unlike *mdt-15* RNAi (Fig. 3A). Moreover, MDT-15’s yeast orthologue Gal11 was identified in a screen for genes involved in oxidative stress sensitivity (Thorpe *et al*., 2004), suggesting that this is an evolutionarily ancient role for MED15 proteins. It will be interesting to test whether mammalian MED15 is required for antioxidant responses, perhaps by coregulating the SKN-1 orthologue Nrf2.

**Figure 6:**
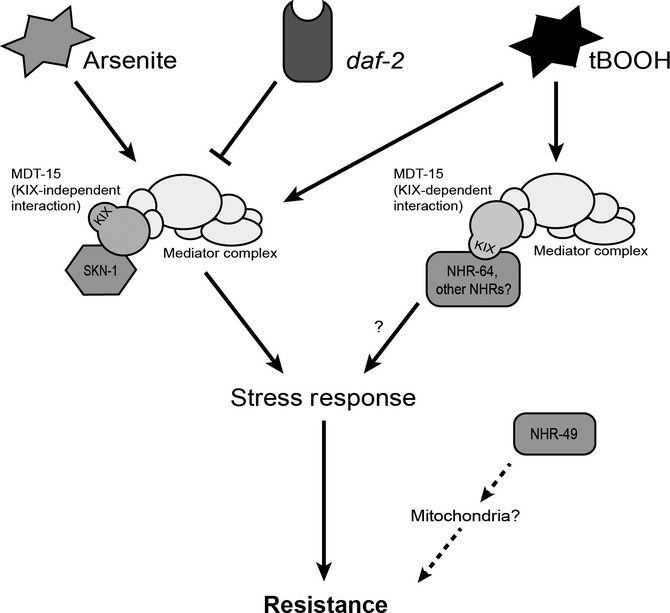
Model of MDT-15 action in the oxidative stress response. In the arsenite response and downstream of *daf-2*, MDT-15 interacts with SKN-1 to regulate some stress response genes such as *gst*s. MDT-15 is also required for the SKN-1-independent tBOOH response, presumably involving NHRs; *nhr-49* might be broadly sensitive to oxidative stress due to its impact on mitochondria.

Arsenite attacks the thiol groups of glutathione and other peptides and promotes ROS production, whereas tBOOH directly damages proteins and lipids. Worms mount distinct defenses against each molecule: SKN-1 is required to induce arsenite response genes, but plays only a minor role in the tBOOH response (Oliveira *et al*., 2009). MDT-15 is essential for both responses, suggesting a broad role in cytoprotective pathways. To implement specific responses, MDT-15 interacts with SKN-1 to combat arsenite exposure and may act with NHR-64 or other NHRs to defend against tBOOH (Fig. 6). Functional RNAi screens using promoter::GFP reporter fusions will be useful to identify the genes required to induce tBOOH responsive genes.

MDT-15 physically binds SKN-1; to our knowledge, this is the first report of a positive coregulator for SKN-1. The interaction is KIX-domain independent, as MDT-15 instead associates with SKN-1c via a region partially deleted in *mdt-15(rf)* mutants. This region is also required for NHR-64 binding, although NHR-64 also requires the KIX-domain. We favor the view that the tm2182 region is directly involved in protein binding rather than causing MDT-15 misfolding, because MDT-15tm2182 is transcriptionally active in yeast suggesting that it adopts functional conformation, is expressed at wild-type levels *in vivo*, and *tm2182* causes hypomorph phenotypes that are less severe than *mdt-15* depletion. A multisurface interaction between MDT-15 and NHR-64 might thus resemble the interaction between Gcn4 and Gal11 in yeast (Herbig *et al*., 2010; Jedidi *et al*., 2010).

Depleting the fatty acid desaturases *fat-6* or *fat-7* causes paraquat sensitivity (Horikawa & Sakamoto, 2009). We found that *mdt-15(rf)* and *nhr-49(−)* mutants, which strongly downregulate these genes and show altered fatty acid profiles (Van Gilst *et al*., 2005; Taubert *et al*., 2006; Yang *et al*., 2006), are also sensitive to oxidative stress. This initially suggested that fatty acid imbalance might cause oxidative stress susceptibility. However, *fat-6(RNAi)* and *fat-6(−); fat-7(−)* worms were insensitive to tBOOH despite pleiotropic phenotypes, and PUFA complementation failed to protect *mdt-15(rf)* mutants from tBOOH. Thus, the altered fatty acid composition of *mdt-15* worms is unlikely to cause oxidative stress sensitivity, although it impacts growth, fertility, and mobility. Regulation of lipid biology and oxidative stress responses are thus separable MDT-15-regulated processes. Viewed more broadly, our data show that even severe changes in lipid composition do not necessarily disrupt stress responses.

Despite not being required for tBOOH resistance, we found that fatty acid desaturases were downregulated by oxidative stress. Synthesizing PUFAs in an oxidizing environment may be undesirable as they are peroxidation targets. Accordingly, we observed that exogenous PUFAs caused slight tBOOH sensitivity in wild-type animals. Alternatively, factors such as MDT-15, which is required to transcribe *fat-5*, *fat-6*, and *fat-7*, may be required to transcribe cytoprotective pathways rather than promote anabolic metabolism in a pro-oxidative environment.

### MDT-15’s role in aging

The view of ROS in aging has evolved in recent years (Hekimi *et al*., 2011; Back *et al*., 2012). Low levels of oxidative stress have been proposed to elicit beneficial stress responses early in life, which may defend against oxidative damage later in life (Hekimi *et al*., 2011; Back *et al*., 2012). Yet, as organisms age, cellular damage accumulates, and the induction of cytoprotective pathways is likely critical to combat age-related molecular damage (Shore *et al*., 2012). Factors such as MDT-15 should thus contribute to the longevity of certain mutants (Hou & Taubert, 2012). Indeed, *mdt-15* is at least partially required for the long lifespan of insulin receptor mutants, dietary-restriction*-*mimicking *eat-2* mutants, the translation pathway mutants *ifg-1* and *rsks-1*, the nutrient sensor mutant *aak-2*, and the germline-less *glp-1* mutant (Rogers *et al*., 2011; McCormick *et al*., 2011; Zhang *et al*., 2013; and this study). However, *mdt-15* mutation or depletion also shortens the lifespan of wild-type worms (Taubert *et al*., 2006). Thus, MDT-15 regulated processes likely counteract aging in many genotypes.

In *daf-2* mutants, DAF-16-driven intestinal *mdt-15* induction was proposed to promote downstream lipid signaling to distant tissues not expressing *mdt-15*, thus coordinating longevity throughout the worm (Zhang *et al*., 2013). However, we observed no *mdt-15* induction in *daf-2(e1370)* worms (Fig. S6D), and Zhang *et al*. found unaltered intestinal expression of *mdt-15p::gfp* in *daf-2* mutants. Even if not induced, MDT-15 could be essential for downstream processes. We propose that MDT-15’s function in ROS defense may also underlie its lifespan promoting role, perhaps in parallel to *mdt-15-*dependent lipid signaling.

Together, our data provide evidence that MDT-15 is required for the defense against two distinct exogenous oxidative stressors. This role of MDT-15 is separable from its impact on fatty acid metabolism, which suggests that altered lipid composition does not strictly correlate with an inability to combat oxidative stress. The broad cytoprotective activities of MDT-15 involve multiple transcription factors and highlight this conserved Mediator subunit as an important contributor to ROS homeostasis.

## Experimental procedures

### Nematode strains, growth conditions, and RNAi

We cultured *C. elegans* strains using standard techniques (Brenner, 1974) and *E. coli* OP50 as food, except for RNAi. For worm strains, see Table S8. To avoid background effects, each mutant was crossed into our N2 strain; original mutants were backcrossed to N2 at least six times. All experiments were carried out at 20 °C, except those with temperature sensitive *daf-2* mutants, which were grown at 16 °C to prevent dauer entry, and shifted to 25 °C for experiments.

RNAi was performed on nematode growth media (NGM) plates with 25 μg mL^−1^ carbenicillin, 1 mm IPTG, and 12.5 μg mL^−1^ tetracycline, and seeded with appropriate HT115 RNAi bacteria. The *mdt-15*, *skn-1*, and *wdr-23* RNAi clones are from the Ahringer library (Kamath *et al*., 2003) and were sequenced prior to use. Sodium meta-arsenite (Sigma 71287), tBOOH (Sigma 458139), oleic acid (S-1120), linoleic acid (S-1127), and eicosapentaenoic acid (S-1144) (Nu-Chek Prep) were added at indicated concentrations.

For synchronized worm growths, bleached embryos were hatched overnight on unseeded NGM plates; hatched, synchronized L1 larvae were then grown to the desired stage, as indicated, and growths were adapted to ensure developmental synchronicity of slow-growing *wdr-23(−)* and *mdt-15(rf)* mutants.

For stress response and lifespan assays, animals were grown to the late L4 stage and then transferred to normal or oxidant-containing plates. We used GraphPad prism 5 (GraphPad Software, Inc., La Jolla, CA, USA) to generate survival curves and calculated statistical significance using the log-rank (Mantel–Cox) test.

### RNA isolation and quantitative PCR analysis

RNA isolation was performed as described (Taubert *et al*., 2006), with an added sonication step of the Trizol suspension to improve RNA yield. 2 μg total RNA was used to generate cDNA with superscript II reverse transcriptase (Invitrogen 18064-014), random primers (Invitrogen 48190-011), dNTPs (Fermentas R0186), and RNAseOUT (Invitrogen 10777-019). qPCR was performed in 30 μL reactions using Taq (Invitrogen 18038-240) and an Applied Biosystems StepOnePlus machine. We analyzed data with the 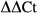 method. For each sample, we calculated normalization factors by averaging the (sample expression)/(reference expression) ratios of three or four normalization genes, as indicated; the reference was *control(RNAi)*, WT, or untreated, as appropriate. We used nonparametric Mann–Whitney tests to calculate statistical significance of gene expression changes. Primers were tested on serial cDNA dilutions and analyzed for PCR efficiency (sequences in Table S9).

### DIC and fluorescence microscopy

Worms were transferred onto 2% (w/v) agarose pads for microscopy. We captured images on a CoolSnap HQ camera (Photometrics, Tucson, AZ, USA) attached to a Zeiss Axioplan 2 compound microscope (Carl Zeiss Microscopy, Thornwood, NY, USA) and used MetaMorph Imaging Software with Autoquant 3D (Molecular Devices, LLC, Sunnyvale, CA, USA) digital deconvolution for image acquisition.

### Yeast-two-hybrid assays and immunoblots

To estimate relative interaction strength, we transformed plasmid pairs into strain Y187 (Clontech, Mountain View, CA, USA) and performed liquid β-galactosidase assays as described (Taubert *et al*., 2006) using an OmegaStar plate reader (BMG Labtech, Ortenberg, Germany). Each assay included at least four technical replicates and was repeated three or more times. Western blots to detect protein expression in yeast and in *C. elegans* were carried out as described (Taubert *et al*., 2006) using Myc (Santa Cruz sc-40, Santa Cruz, CA, USA), Gal4AD (Clontech 630402, Mountain View, CA, USA), β-actin (Cell Signaling Technologies, Danvers, MA, USA), and MDT-15 (Taubert *et al*., 2006) antibodies.

## Supporting Information

**Fig. S1** SKN-1 and DAF-16 expression and localization are unaffected by *mdt-15* depletion or mutation. **Fig. S2** *mdt-15* is required for arsenite gene inductions in L4 and adult worms. **Fig. S3** MDT-15 is required to upregulate SKN-1 targets upon loss of *wdr-23*. **Fig. S4** Expression controls for yeast-two-hybrid fusion proteins. **Fig. S5** The *tm2182* mutation is an in-frame deletion and the resulting MDT-15 protein is expressed *in vivo* and transcriptionally active in yeast. **Fig. S6** Requirement of *mdt-15* for gene expression and longevity in two *daf-2* mutants. **Fig. S7** *fat-6(tm331); fat-7(wa36)* double mutants are not sensitive to tBOOH and *fat-6* RNAi causes developmental defects. **Fig. S8** *mdt-15* is required to induce several tBOOH responsive genes.

**Table S1** Identities of genes downregulated in *mdt-15(RNAi)* worms and induced by oxidative stress. **Table S2** Survival of mutant strains on 5 mM sodium arsenite. **Table S3** Survival of mutant strains on 6 mM tBOOH. **Table S4** Survival of RNAi-treated worms on 6 mM tBOOH. **Table S5** Survival of PUFA-treated worms on 6 mM tBOOH. **Table S6** Lifespans of *daf-2(e1370)* mutants on control and *mdt-15* RNAi. **Table S7** Lifespans of *daf-2(e1368)* mutants on control and *mdt-15* RNAi. **Table S8** List of worm strains. **Table S9** List of qPCR primers.
