# Supplementary material for: The conserved Mediator subunit MDT-15 is required for oxidative stress responses in Caenorhabditis elegans
Source: Aging Cell. 2013 Sep 18;13(1):70–9. doi: 10.1111/acel.12154 (PMC4326869; doi:10.1111/acel.12154)

Fig. S1. *skn-1* and *daf-16* expression and DAF-16 localization are unaffected by *mdt-15* depletion or mutation.
(A) mRNA fold change of *skn-1* and *daf-16* in *mdt-15(RNAi)* worms relative to *control(RNAi)* (n=4). mRNA levels were normalized to *act-1*, *ama-1*, *cdc-42*, and *tba-1*; error bars represent SEM. (B) Same as (A) but with N2 and *mdt-15(rf)* worms. *p<0.05. (C) Fluorescence micrographs showing DAF-16::GFP expression in *control(RNAi)* and *mdt-15(RNAi)* worms, with or without acute sodium arsenite treatment. (D) Fluorescence micrographs showing SKN-1::GFP expression in *control(RNAi)* and *mdt-15(RNAi)* worms, both also treated with *wdr-23(RNAi)*. Autofluorescence is shown in the TxRed channel (magenta).

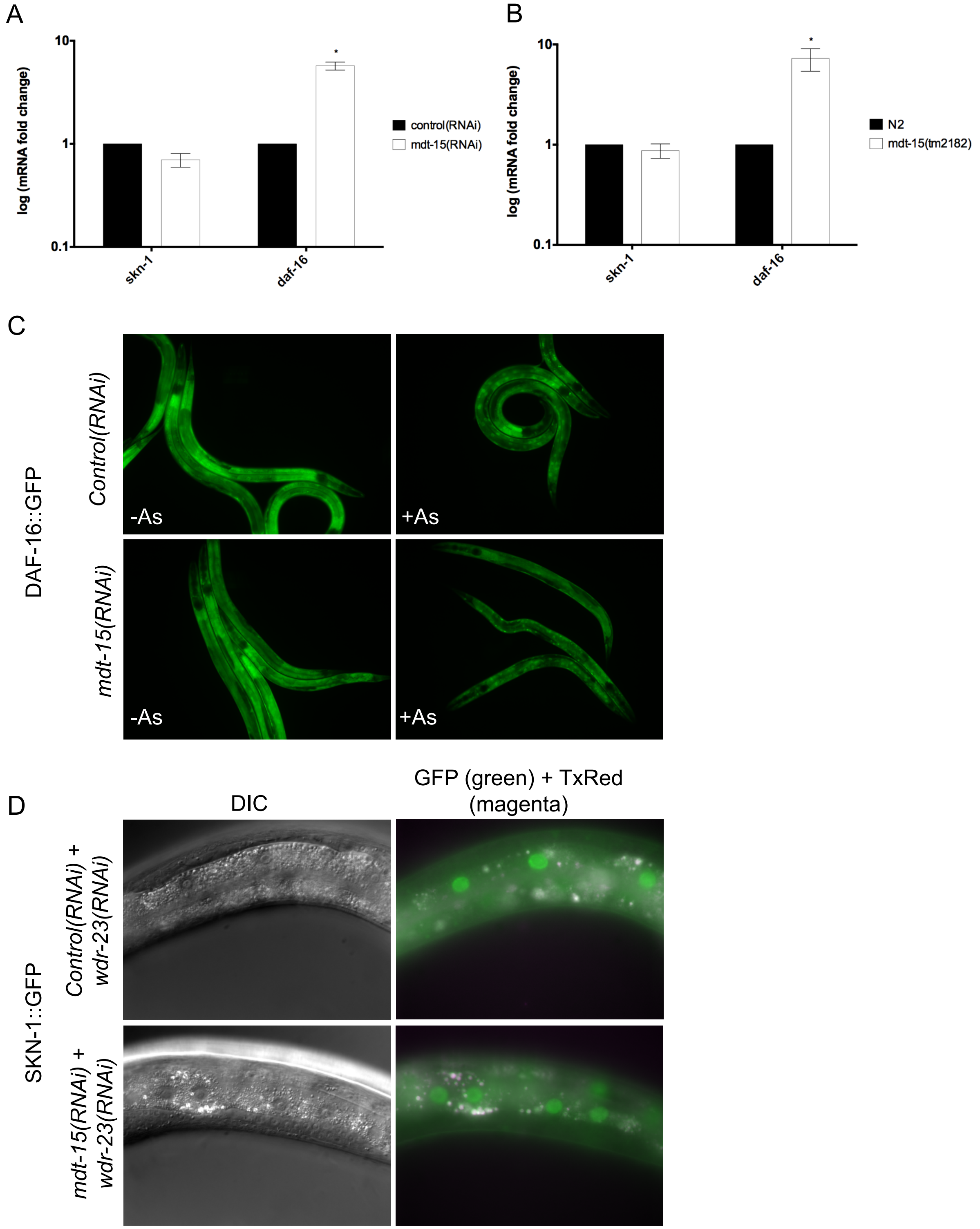

Supplementary Figure 2

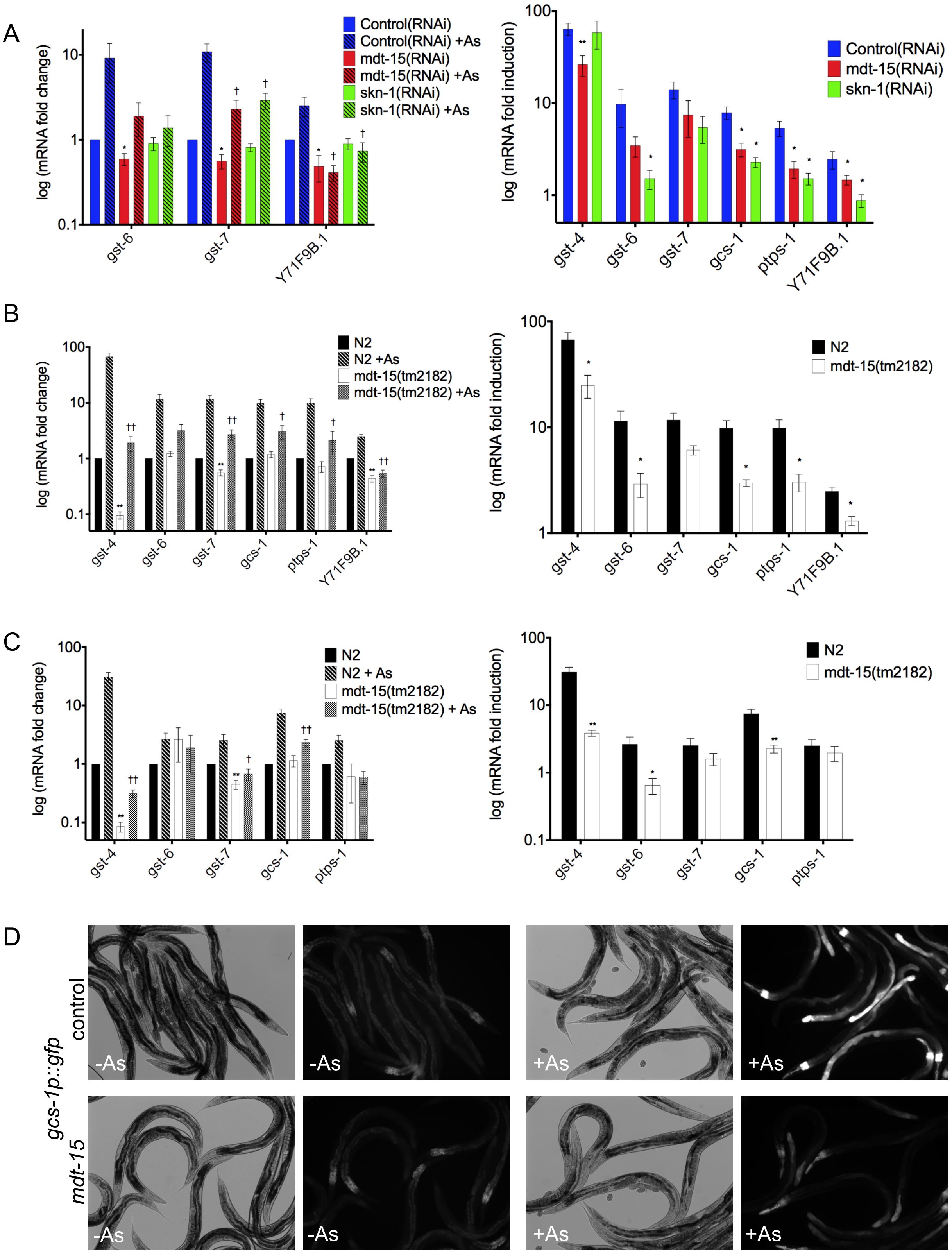

Fig. S2. *mdt-15* is required for arsenite gene inductions in L4 and adult worms.

(A) Bar graphs depict mRNA fold changes of additional arsenite-responsive genes tested in control, *mdt-15* and *skn-1* RNAi worms, relative to untreated *control(RNAi)* (see Fig. 2A). mRNA levels were normalized to *act-1*, *ama-1*, *cdc-42* and *tba-1*; error bars represent SEM. *Untreated worms differ significantly from *control(RNAi)* ($p<0.05$); †Treated worms differ significantly from *control(RNAi)* ($p<0.05$). Fold inductions of all arsenite-responsive genes are also shown (*$p<0.05$).

(B) mRNA fold changes (relative to untreated N2) in N2 and *mdt-15(rf)* worms exposed acutely to 5 mM As (n=5). mRNA levels were normalized to *act-1*, *ama-1*, *cdc-42* and *tba-1*; error bars represent SEM. **Untreated mutants differ significantly from N2 ($p<0.01$). †,†† Treated mutants differ significantly from N2 ($p<0.05$ and $p<0.01$ respectively). Fold inductions are also shown. *$p<0.05$.

(C) mRNA fold changes (relative to untreated N2) in N2 and *mdt-15(rf)* worms exposed to 5 mM As for one hour (n=5). mRNA levels were normalized to *act-1*, *tba-1* and *ubc-2*; error bars represent SEM. **Untreated mutants differ significantly from N2 ($p<0.01$). †,†† Treated mutants differ significantly from N2 ($p<0.05$ and $p<0.01$ respectively). Fold inductions are also shown. *,**$p<0.05$ and $p<0.01$ respectively.

(D) Fluorescence micrographs showing *gcs-1p::gfp* worms grown to the L4 stage then treated with control or *mdt-15* RNAi for 2 days. They were then either left untreated or acutely exposed to 5 mM arsenite.

Fig. S3. MDT-15 is required to upregulate SKN-1 targets upon *wdr-23* loss of function.
(A) Fold changes of additional SKN-1 targets in N2 and *wdr-23(-)* worms grown on control, *mdt-15*, *skn-1* and *mdt-6* RNAi, relative to *control(RNAi)* (n=4) (see Fig. 3A). (B) Fold changes of SKN-1 targets in N2 and *mdt-15(rf)* worms grown on control and *wdr-23* RNAi, relative to *control(RNAi)* (n=4). For all experiments mRNA levels were normalized to *act-1*, *ama-1*, *cdc-42* and *tba-1*; error bars represent SEM. *p<0.05.

A

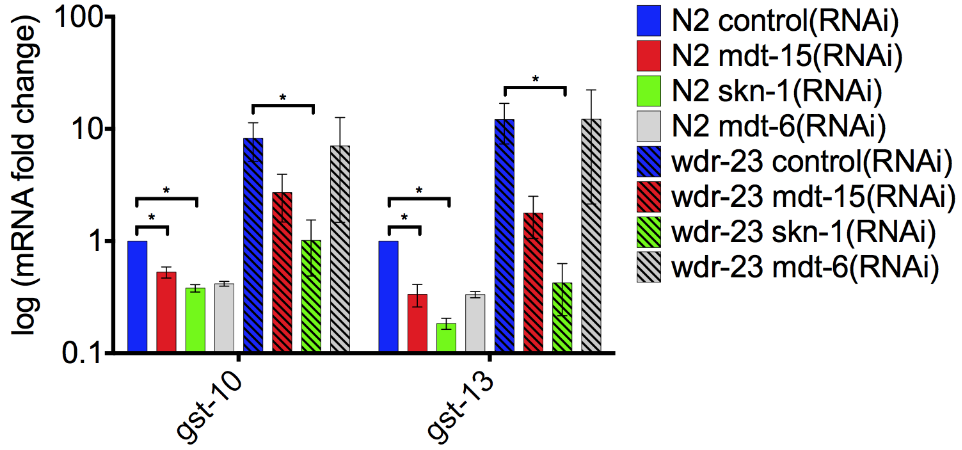

B

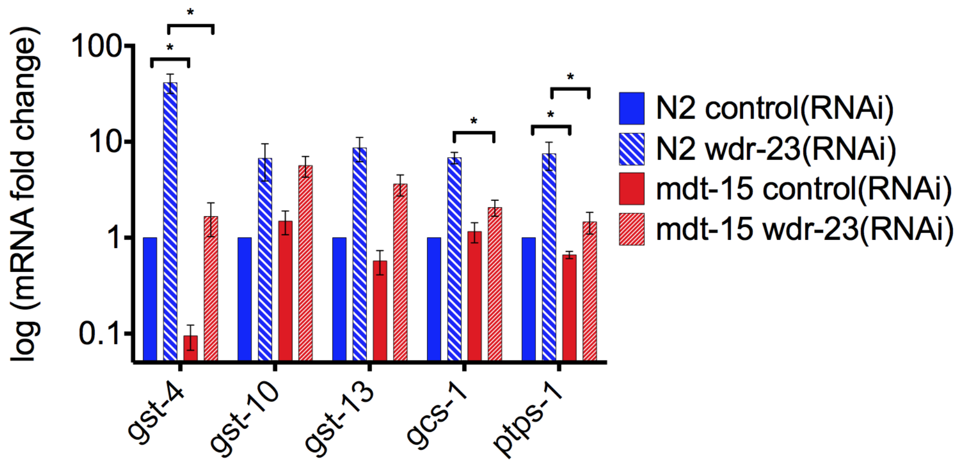

Fig. S4. Expression controls for yeast-two-hybrid fusion proteins.
The Western blot shows the expression of Gal4DBD::MDT-15 variants used in the yeast-two-hybrid analysis (see Fig. 3B). Asterisks indicate the expected size of individual fusion proteins.

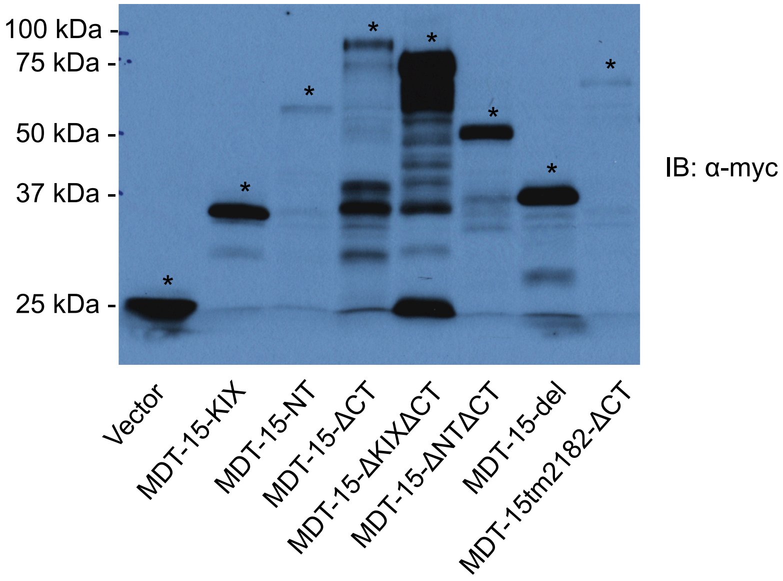

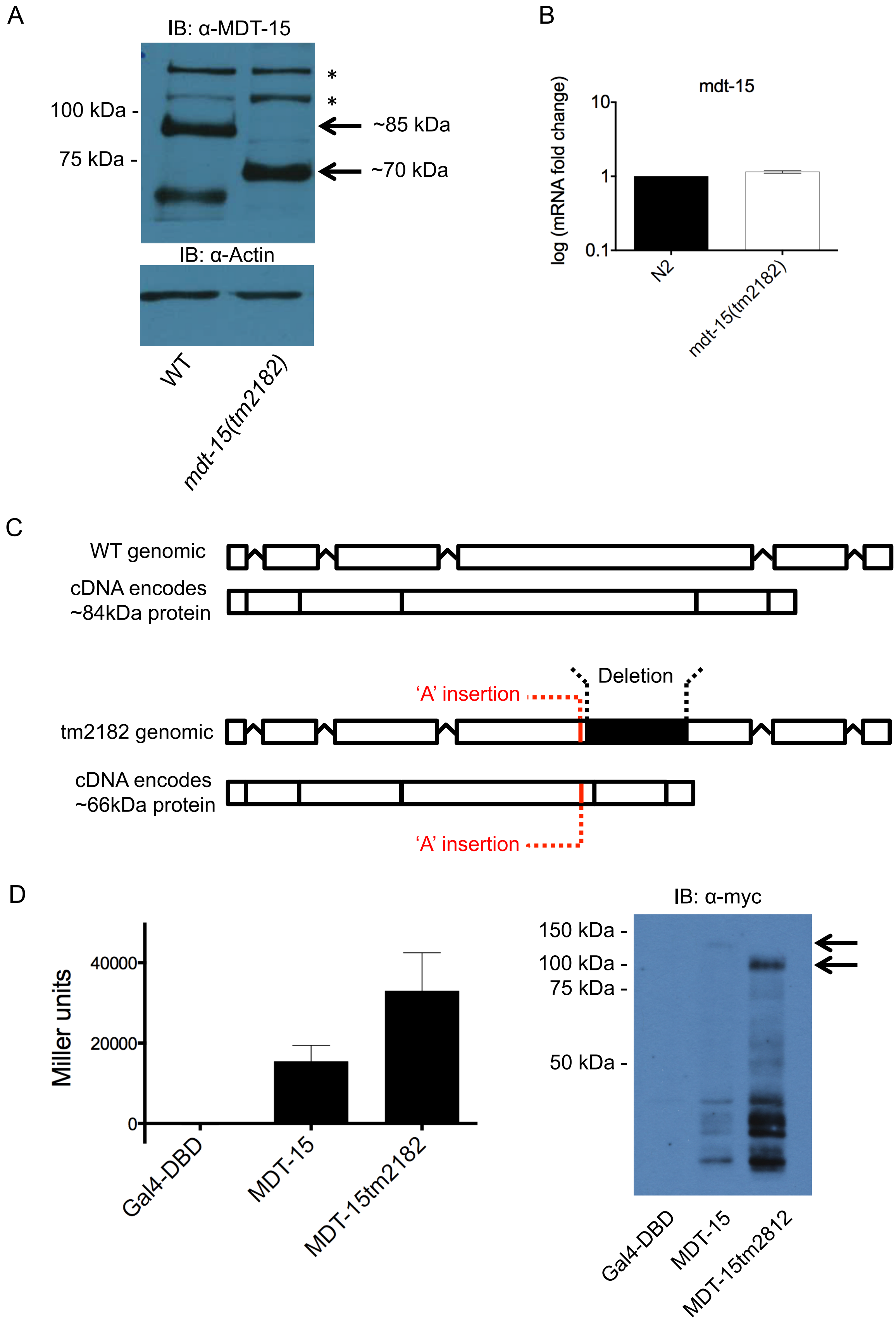
A
IB: α-MDT-15
100 kDa -
75 kDa -
*
*
~85 kDa
~70 kDa
IB: α-Actin
WT
mdt-15(tm2182)
B
mdt-15
log (mRNA fold change)
10
1
0.1
N2
mdt-15(tm2182)
C
WT genomic
cDNA encodes
~84kDa protein
Deletion
'A' insertion
tm2182 genomic
cDNA encodes
~66kDa protein
'A' insertion
D
Miller units
40000
20000
0
Gal4-DBD
MDT-15
MDT-15tm2182
IB: α-myc
150 kDa -
100 kDa -
75 kDa -
50 kDa -
Gal4-DBD
MDT-15
MDT-15tm2812

Fig. S5. The *tm2182* mutation is an in-frame deletion and the resulting MDT-15 protein is expressed *in vivo* and transcriptionally active in yeast. (A) Immunoblot analysis shows the ~70kDa MDT-15 protein produced in *mdt-15(rf)* mutants, compared to the ~85kDa WT protein. Actin was used as a loading control. The bands indicated with asterisks (*) represent non-specific bands. (B) The bar graphs depict the mRNA fold change of *mdt-15* transcript levels in the *mdt-15(rf)* mutant relative to wild-type N2 worms (n=7). mRNA levels were normalized to *act-1*, *ama-1*, *cdc-42* and *tba-1*; error bars represent SEM. (C) Schematic detailing the nature of the in-frame *tm2182* mutation, which causes a 161 aa deletion and a 2 aa insertion. The 'A' insertion just upstream of the deletion restores the reading frame. (D) Bar graphs show estimates of the activation capacity of the Gal4DBD, Gal4DBD-MDT-15, and Gal4DBD-MDT-15tm2182 fusion proteins using a Gal4-UAS driven β-galactosidase activity assay. Error bars represent SEM. The Western blot shows expression of the fusion proteins.

Fig. S6. Requirement of *mdt-15* for gene expression and longevity of two *daf-2* mutants.
(A) DIC and fluorescence micrographs show worms expressing *sod-3p::gfp* in a *daf-2(e1370)* background treated with control or *mdt-15* RNAi. (B) Bar graphs depict additional mRNA fold changes in N2 and *daf-2(e1370)* worms grown on control, *mdt-15*, or *skn-1* RNAi (n=4) (see Fig. 3C). (C) mRNA fold change of *mdt-15* in *daf-2(e1370)* worms relative to N2 (n=4). For (B) and (C) mRNA levels were normalized to *act-1*, *ama-1*, *cdc-42* and *tba-1*; error bars represent SEM. *p<0.05. (D) Lifespan assay of N2 and *daf-2(e1370)* worms grown on control and *mdt-15* RNAi. For details, see Table S5. (E) Same experiment as in (D), but with *daf-2(e1368)* mutants. For details, see Table S6.

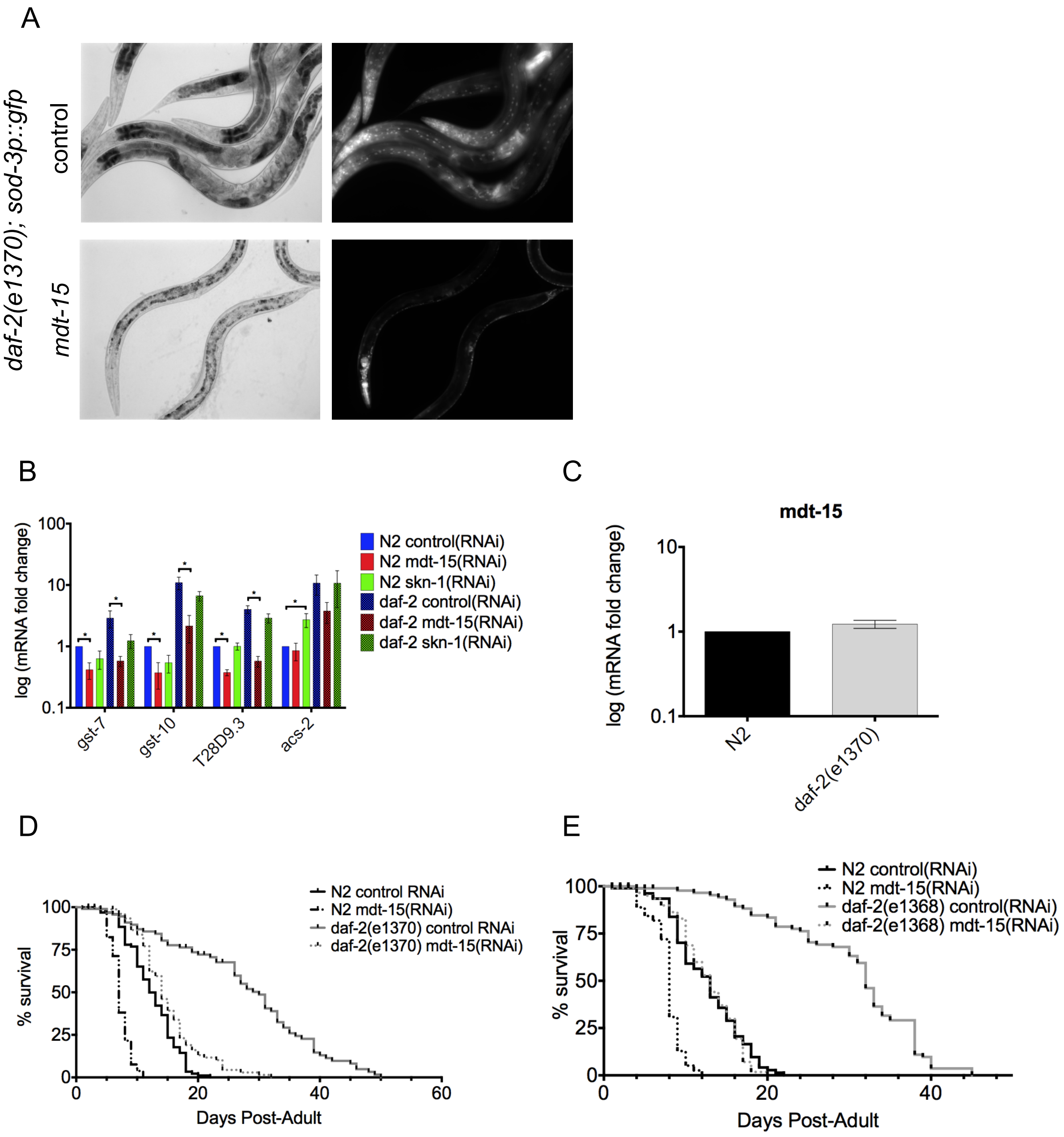

Fig. S7. *fat-6(tm331); fat-7(wa36)* double mutants are not sensitive to tBOOH and *fat-6* RNAi causes developmental defects.
(A) Survival plots of N2 and *fat-6(tm331); fat-7(wa36)* worms on 6 mM tBOOH. One representative experiment out of three independent repeats is shown. For details see Table S2. (B) DIC micrographs of N2 worms grown to the L4 stage on control, *mdt-15* and *fat-6* RNAi.

A

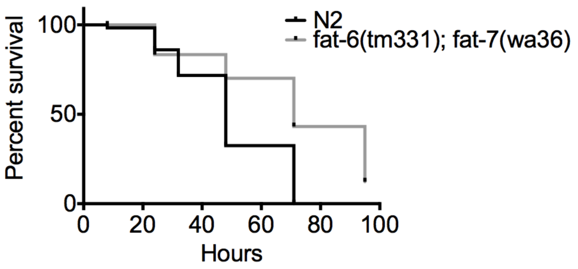

B

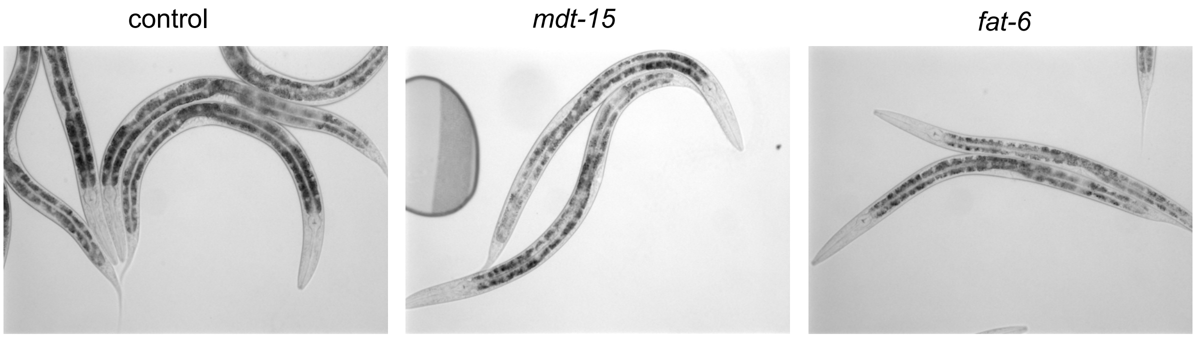

Fig. S8. *mdt-15* is required to induce several tBOOH responsive genes.
(A) mRNA fold changes of additional tBOOH-responsive genes tested in control, *mdt-15* and *skn-1* RNAi worms, relative to untreated *control(RNAi)* worms (see Fig. 5A). mRNA levels were normalized to *act-1*, *ama-1*, *cdc-42* and *tba-1*; error bars represent SEM. *Untreated worms differ significantly from *control(RNAi)* (p<0.05); †Treated worms differ significantly from *control(RNAi)* (p<0.05). Fold inductions of all tBOOH-responsive genes are also shown (*p<0.05). (B) Fold changes in tBOOH responsive genes in N2 and *mdt-15(rf)* worms exposed for four hours to 7.5 mM tBOOH, relative to untreated N2 worms (n=4). mRNA levels were normalized to *act-1*, *ama-1*, *cdc-42* and *tba-1*; error bars represent SEM. *Untreated *mdt-15(tm2182)* worms differ significantly from N2 worms (p<0.05). †Treated *mdt-15(tm2182)* worms differ significantly from N2 worms (p<0.05). Fold inductions from are also shown (*p<0.05). (C) mRNA fold changes (relative to untreated N2) in N2 and *mdt-15(rf)* worms exposed to 7.5 mM tBOOH for one hour (n=5). mRNA levels were normalized to *act-1*, *tba-1* and *ubc-2*; error bars represent SEM. **Untreated mutants differ significantly from N2 (p<0.01). †,†† Treated mutants differ significantly from N2 (p<0.05 and p<0.01 respectively). Fold inductions are also shown (*,**p<0.05 and p<0.01 respectively).

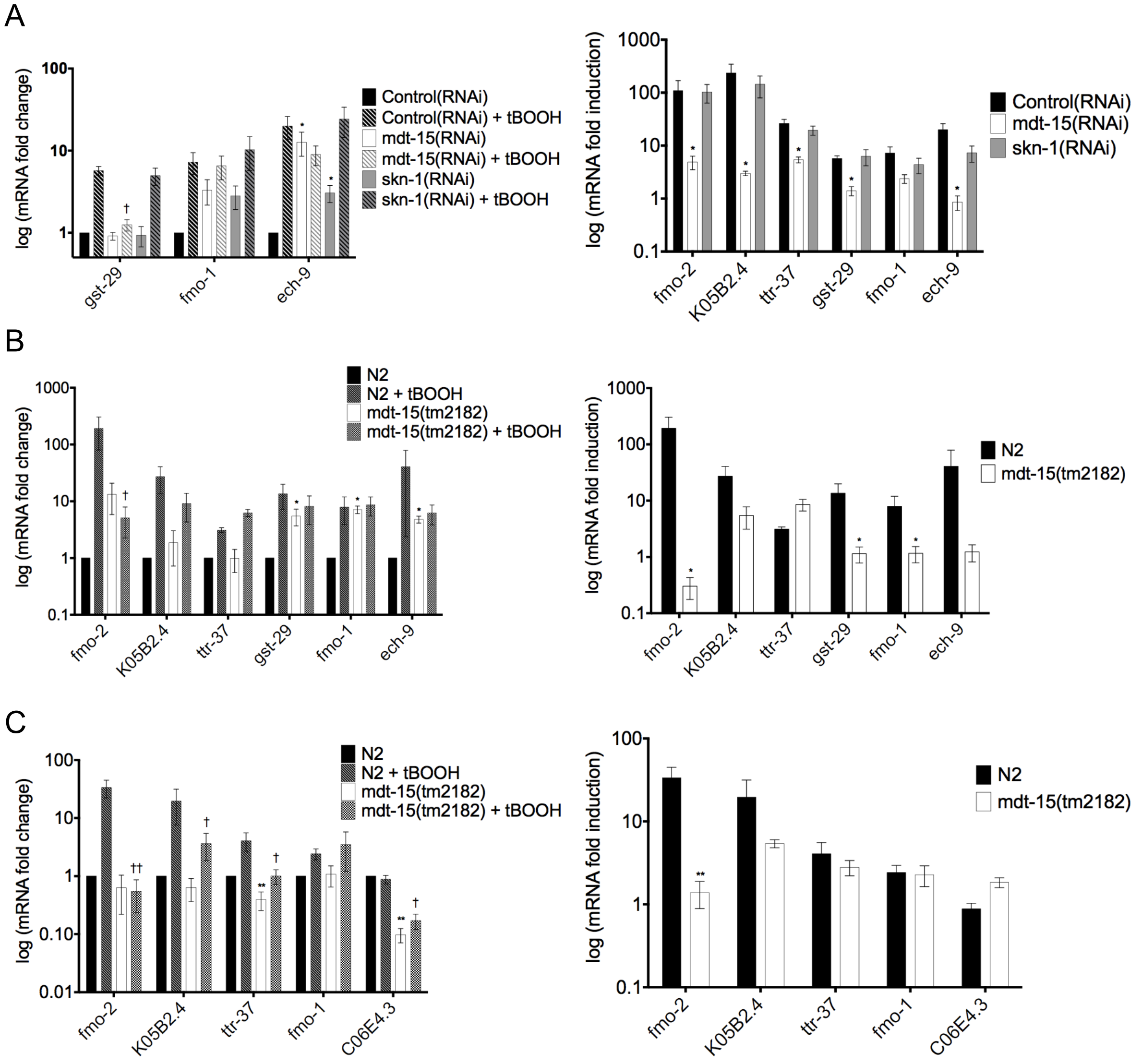

Supplement: Supplementary file 1 — Fig. S1 SKN-1 and DAF-16 expression and localization are unaffected by mdt-15 depletion or mutation. Fig. S2 mdt-15 is required for arsenite gene inductions in L4 and adult worms. Fig. S3 MDT-15 is required to upregulate SKN-1 targets upon loss of wdr-23. Fig. S4 Expression controls for yeast-two-hybrid fusion proteins. Fig. S5 The tm2182 mutation is an in-frame deletion and the resulting MDT-15 protein is expressed in vivo and transcriptionally active in yeast. Fig. S6 Requirement of mdt-15 for gene expression and longevity in two daf-2 mutants. Fig. S7 fat-6(tm331); fat-7(wa36) double mutants are not sensitive to tBOOH and fat-6 RNAi causes developmental defects. Fig. S8 mdt-15 is required to induce several tBOOH responsive genes. [file acel0013-0070-sd1.pdf]
