# Supplementary material for: The conserved Mediator subunit MDT-15 is required for oxidative stress responses in Caenorhabditis elegans
Source: Aging Cell. 2013 Sep 18;13(1):70–9. doi: 10.1111/acel.12154 (PMC4326869; doi:10.1111/acel.12154)
Supplement: Supplementary file 2 — Table S1 Identities of genes downregulated in mdt-15(RNAi) worms and induced by oxidative stress. Table S2 Survival of mutant strains on 5 mM sodium arsenite. Table S3 Survival of mutant strains on 6 mM tBOOH. Table S4 Survival of RNAi-treated worms on 6 mM tBOOH. Table S5 Survival of PUFA-treated worms on 6 mM tBOOH. Table S6 Lifespans of daf-2(e1370) mutants on control and mdt-15 RNAi. Table S7 Lifespans of daf-2(e1368) mutants on control and mdt-15 RNAi. Table S8 List of worm strains. Table S9 List of qPCR primers. [file acel0013-0070-sd2.docx]

**Supplementary Tables**

**Table S1: Identity of overlapping genes between *mdt-15* RNAi dataset and oxidative-stress-induced genes**

|  | **Sequence name** | **Gene name** |
| --- | --- | --- |
| Arsenite-induced (Oliveira et al., 2009) | B0041.6 | *ptps-1* |
|  | F11G11.1 | *gst-8* |
|  | F11G11.2 | *gst-7* |
|  | F11G11.3 | *gst-6* |
|  | F25D1.5 |  |
|  | F35H8.6 | *ugt-58* |
|  | F46C5.10 |  |
|  | R03D7.6 | *gst-5* |
|  | T26C5.1 | *gst-13* |
|  | Y37A1B.5 |  |
|  | Y71F9B.1 |  |
| tBOOH-induced (Oliveira et al., 2009) | C05E4.9 | *icl-1* |
|  | F11G11.1 | *gst-8* |
|  | F11G11.2 | *gst-7* |
|  | F21E9.3 | *ttr-37* |
|  | F32D8.12 |  |
|  | F47B8.2 |  |
|  | F59B1.8 |  |
|  | R03D7.6 | *gst-5* |
|  | ZK550.6 |  |
| O2-induced (Park et al., 2009) | C01G6.7 | *acs-7* |
|  | C05C10.3 |  |
|  | C05E4.9 | *icl-1* |
|  | C10H11.3 | *ugt-25* |
|  | C10H11.6 | *ugt-26* |
|  | C29E6.5 | *nhr-43* |
|  | C29F9.3 |  |
|  | C39E9.8 |  |
|  | F01D5.2 |  |
|  | F11G11.3 | *gst-6* |
|  | F14E5.5 | *lips-10* |
|  | F17C11.6 |  |
|  | F18E3.7 | *ddo-2* |
|  | F23F12.12 |  |
|  | F28A10.6 | *acdh-9* |
|  | F32D8.12 |  |
|  | F35H8.6 | *ugt-58* |
|  | F36A2.3 |  |
|  | F37B4.7 | *folt-2* |
|  | F47B8.2 |  |
|  | F58F9.7 |  |
|  | F58G6.2 | *srm-3* |
|  | H12C20.3 | *nhr-68* |
|  | H23N18.1 | *ugt-13* |
|  | M02D8.4 | *asns-2* |
|  | M03A8.1 | *dhs-28* |
|  | M88.1 | *ugt-62* |
|  | R03D7.6 | *gst-5* |
|  | T21E8.2 | *pgp-7* |
|  | T26C5.1 | *gst-13* |
|  | W02A2.1 | *fat-2* |
|  | W02H5.8 |  |
|  | Y32F6B.1 |  |
|  | ZC455.6 | *ugt-5* |
|  | ZK1320.9 |  |
|  | ZK550.6 |  |
|  | ZK945.1 | *lact-2* |
| Juglone-induced in L4 (Przybysz et al., 2009) | B0041.6 | *ptps-1* |
|  | C04F5.7 | *ugt-63* |
|  | C29F3.7 |  |
|  | F11G11.2 | *gst-7* |
|  | F31F7.1 |  |
|  | H23N18.1 | *ugt-13* |
|  | R03D7.6 | *gst-5* |
|  | T26C5.1 | *gst-13* |
|  | W06D12.3 | *fat-5* |
|  | Y38E10A.13 | *nspe-1* |
| SKN-1 induced (Oliveira et al., 2009) | AC3.7 | *ugt-1* |
|  | B0041.6 | *ptps-1* |
|  | C29F7.2 |  |
|  | F08G5.6 |  |
|  | F10D2.9 | *fat-7* |
|  | F11G11.1 | *gst-8* |
|  | F11G11.2 | *gst-7* |
|  | F11G11.3 | *gst-6* |
|  | F25D1.5 |  |
|  | R151.2 |  |
|  | T21E8.2 | *pgp-7* |
|  | T26C5.1 | *gst-13* |
|  | Y37A1B.5 |  |

For Table S2-6, number of subjects is denoted as follows: Dx=Number of deaths that occurred during the assay; Nx=Total number of animals used in assay; Cx=Number of censored events (*i.e.* worms that ruptured at the vulva, underwent internal hatching of the progeny, or crawled off the plate). Where the mean lifespan is given as ND, this indicates that not all animals were dead when the assay was concluded and therefore mean lifespan could not be calculated. All *p*-values are derived using the log-rank (Mantel-Cox) test. **p*<0.05, ***p*<0.01, ****p*<0.001.

**Table S2: Survival of mutant strains on 5 mM sodium arsenite.**

| Strain | Experiment no. | Median lifespan (hours) | Number of subjects (Dx/Nx (Cx)) | *p* value vs. N2 |
| --- | --- | --- | --- | --- |
| N2 | 1 | >100 | 3/107 (0) | NA |
|  | 2 (see Fig. 5B, D) | 100 | 90/105 (5) | NA |
|  | 3 (see Fig. 1A) | >100 | 45/107 (1) | NA |
|  | 4 | 123 | 63/112 (49) | NA |
| *mdt-15(tm2182)* | 1 | 24 | 104/105 (1) | <0.0001*** |
|  | 2 | 24 | 98/100 (2) | <0.0001*** |
|  | 3 (see Fig. 1A) | 24 | 105/105 (0) | <0.0001*** |
| *nhr-49(nr2041)* | 1 | >100 | 29/96 (3) | <0.0001*** |
|  | 2 (see Fig. 5D) | 24 | 90/95 (1) | <0.0001*** |
|  | 3 | 24 | 50/65 (2) | <0.0001*** |
| *nhr-64(ok1957)* | 1 | >100 | 17/101 (3) | 0.0006*** |
|  | 2 (see Fig. 5B) | 100 | 79/99 (13) | 0.1967 |
|  | 3 | 96 | 59/102 (5) | 0.0674 |
|  | 4 | 123 | 77/107 (30) | 0.011* |

**Table S3: Survival of mutant strains on 6 mM tBOOH.**

| Strain | Experiment no. | Median lifespan (hours) | Mean lifespan (hours) ± SEM | Number of subjects (Dx/Nx (Cx)) | *p* value vs. N2 |
| --- | --- | --- | --- | --- | --- |
| N2 | 1 | 48 | 41.02 ± 1.08 | 84/114 (28) | NA |
|  | 2 (see Fig. 1A, 2C) | 35 | 38.78 ± 1.31 | 83/111 (28) | NA |
|  | 3 | 48 | 41.34 ± 2.01 | 84/106 (19) | NA |
|  | 4 (see Fig. 5E) | 49 | 38.79 ± 1.67 | 67/105 (38) | NA |
|  | 5 (see Fig. S7A) | 48 | 42.69 ± 1.65 | 80/121 (41) | NA |
|  | 6 | 97 | ND | 60/116 (51) | NA |
|  | 7 | 36 | 34.70 ± 1.13 | 83/100 (17) | NA |
|  | 8 | 71 | 42.24 ± 4.23 | 45/120 (75) | NA |
| *mdt-15(tm2182)* | 1 | 24 | 18.85 ± 0.68 | 104/105 (1) | <0.0001*** |
|  | 2 (see Fig. 1A) | 10 | 17.06 ± 1.87 | 17/17 (0) | <0.0001*** |
|  | 3 | 24 | 22.72 ± 0.46 | 102/105 (3) | <0.0001*** |
|  | 4 | 24 | 22.74 ± 0.57 | 76/76 (0) | <0.0001*** |
| *nhr-49(nr2041)* | 1 | 24 | 28.37 ± 0.84 | 92/110 (18) | <0.0001*** |
|  | 2 | 25 | 25.19 ± 1.04 | 81/95 (14) | <0.0001*** |
|  | 4 (see Fig. 5E) | 25 | 32.99 ± 1.37 | 80/80 (0) | <0.0001*** |
| *nhr-64(ok1957)* | 1 | 34 | 33.28 ± 1.19 | 59/107 (48) | <0.0001*** |
|  | 2 (see Fig. 5C) | 25 | 27.25 ± 1.27 | 84/129 (45) | <0.0001*** |
|  | 3 | 33 | 32.66 ± 1.20 | 87/116 (29) | 0.0005*** |
| *fat-6(tm331); fat-7 (wa36)* | 5 (see Fig. S7A) | 71 | ND | 17/26 (9) | 0.0002*** (long-lived) |
|  | 6 | 97 | ND | 17/37 (8) | 0.0198* (long-lived) |
|  | 7 | 48 | 42.64 ± 1.70 | 56/85 (29) | <0.0001*** (long-lived) |
|  | 8 | 73 | ND | 61/86 (25) | <0.0001*** (long-lived) |

**Table S4: Survival of RNAi-treated worms on 6 mM tBOOH.**

| RNAi treatment | Experiment no. | Median lifespan (hours) | Mean lifespan (hours) ± SEM | Number of subjects (Dx/Nx (Cx)) | *p* value vs. control |
| --- | --- | --- | --- | --- | --- |
| Control | 1 | 32 | 32.06 ± 1.20 | 85/117 (32) | NA |
|  | 2 (see Fig. 4B) | 71 | 40.80 ± 2.52 | 35/92 (57) | NA |
|  | 3 | 48 | 32.51 ± 2.44 | 37/92 (55) | NA |
| *mdt-15* | 1 | 24 | 23.24 ± 0.43 | 105/110 (5) | <0.0001 |
|  | 2 (see Fig. 4B) | 24 | 24.78 ± 0.25 | 92/97(5) | <0.0001 |
|  | 3 | 24 | 25.05 ± 1.00 | 78/100 (22) | <0.0001 |
| *fat-6* | 1 | 47 | 44.67 ± 1.78 | 97/105 (8) | 0.0052** (long-lived) |
|  | 2 (see Fig. 4B) | 49 | 51.40 ± 2.85 | 59/89 (29) | 0.3144 |
|  | 3 | 48 | 43.56 ± 2.73 | 41/86 (45) | 0.6618 |

**Table S5: Survival of PUFA-treated worms on 6 mM tBOOH.**

| Strain | Experiment no. | PUFA treatment | Median lifespan (hours) | Mean lifespan (hours) ± SEM | Number of subjects (Dx/Nx (Cx)) | *p* value vs. no treatment |
| --- | --- | --- | --- | --- | --- | --- |
| N2 | 1 (see Fig. 4B) | No | 48 | 39.03 ± 1.52 | 86/112 (26) | NA |
|  | 2 |  | 47 | 37.91 ± 2.06 | 56/102 (46) | NA |
|  | 3 |  | 48 | 41.68 ± 1.50 | 76/100 (24) | NA |
|  | 1 (see Fig. 4B) | Yes | 33 | 32.63 ± 1.24 | 80/109 (29) | 0.0166* |
|  | 2 |  | 24 | 27.88 ± 1.22 | 85/109 (24) | <0.0001*** |
|  | 3 |  | 33 | 30.32 ± 1.33 | 76/93 (17) | <0.0001*** |
| *mdt-15(tm2182)* | 1 (see Fig. 4B) | No | 24 | 21.99 ± 1.72 | 70/73 (3) | NA |
|  | 2 |  | 24 | 22.11 ± 0.83 | 47/52 (5) | NA |
|  | 3 |  | 24 | 21.36 ± 0.77 | 67/68 (1) | NA |
|  | 1 (see Fig. 4B) | Yes | 24 | 22.67 ± 0.72 | 79/81 (2) | 0.1687 |
|  | 2 |  | 24 | 22.30 ± 1.01 | 64/81 (17) | 0.3539 |
|  | 3 |  | 24 | 23.35 ± 0.49 | 83/84 (1) | 0.0345* |

**Table S6: Lifespans of *daf-2(e1370)* mutants on control and *mdt-15* RNAi.**

| Strain | Experiment no. | RNAi treatment | Median lifespan (days) | Mean lifespan (days) ± SEM | Number of subjects (Dx/Nx (Cx)) | Decrease in mean lifespan (%) | *p* value vs. control RNAi |
| --- | --- | --- | --- | --- | --- | --- | --- |
| N2 | 1 | Control | 12 | 11.47 ± 0.48 | 79/100 (21) | NA | NA |
|  | 2 (see Fig. S6B) |  | 12 | 12.38 ± 0.42 | 92/100 (8) | NA | NA |
|  | 1 | *mdt-15* | 6 | 6.08 ± 0.25 | 49/100 (51) | 46.99 | <0.0001*** |
|  | 2 (see Fig. S6B) |  | 7 | 7.15 ± 0.19 | 81/100 (19) | 42.25 | <0.0001*** |
| *daf-2(e1370)* | 1 | Control | 25 | 26.73 ± 1.41 | 93/100 (7) | NA | NA |
|  | 2 (see Fig. S6B) |  | 30 | 26.48 ± 1.54 | 67/100 (33) | NA | NA |
|  | 1 | *mdt-15* | 15 | 15.53 ± 0.73 | 83/100 (17) | 41.90 | <0.0001*** |
|  | 2 (see Fig. S6B) |  | 14 | 14.90 ± 0.62 | 73/100 (27) | 43.73 | <0.0001*** |

**Table S7: Lifespans of *daf-2(e1368)* mutants on control and *mdt-15* RNAi.**

| Strain | Experiment no. | RNAi treatment | Median lifespan (days) | Mean lifespan (days) ± SEM | Number of subjects (Dx/Nx (Cx)) | Decrease in mean lifespan (%) | *p* value vs. control RNAi |
| --- | --- | --- | --- | --- | --- | --- | --- |
| N2 | 1 (see Fig. S6C) | Control | 13 | 12.58 ± 0.51 | 73/100 (27) | NA | NA |
|  | 2 |  | 12 | 11.42 ± 0.50 | 72/100 (28) | NA | NA |
|  | 1 (see Fig. S6C) | *mdt-15* | 8 | 7.56 ± 0.33 | 41/100 (59) | 39.90 | <0.0001*** |
|  | 2 |  | 8 | 7.85 ± 0.18 | 72/100 (28) | 31.26 | <0.0001*** |
| *daf-2(e1368)* | 1 (see Fig. S6C) | Control | 32 | 30.05 ± 0.98 | 83/100 (17) | NA | NA |
|  | 2 |  | 27 | 24.67 ± 0.87 | 83/97 (14) | NA | NA |
|  | 1 (see Fig. S6C) | *mdt-15* | 13 | 12.43 ± 0.54 | 58/100 (42) | 50.59 | <0.0001*** |
|  | 2 |  | 12 | 12.19 ± 0.47 | 85/100 (15) | 58.64 | <0.0001*** |

**Table S8: List of primers used in qPCR experiments**

| Gene | Forward primer | Reverse primer |
| --- | --- | --- |
| *gst-4* | gatgctcgtgctcttgctg | ccgaattgttctccatcgac |
| *gst-6* | TTTGGCAGTTGTTGAGGAG | TGGGTAATCTGGACGGTTTG |
| *gst-7* | ggacaacagaatcccaaagg | gtaacgggcgatagcatgag |
| *gcs-1* | AATCGATTCCTTTGGAGACC | ATGTTTGCCTCGACAATGTT |
| *ptps-1* | tggtgtatgacctggcaaag | cggatttcagcttctcgaac |
| Y71F9B.1 | TTTGGGCCTTCTGGCTTAC | CGATGGAGAGGGATGAGAG |
| *gst-10* | GTCTACCACGTTTTGGATGC | ACTTTGTCGGCCTTTCTCTT |
| *fat-5* | caactaccatcacaccttcc | cccgttcagtttcacagcc |
| *fat-6* | caacttccatcacacattccc | tcctcgttgaatatcacatcc |
| *acs-2* | agtgagacttgacagttccg | cttgtaagagaggaatggctc |
| *sod-3* | GCTGCAATCTACTGCTCGCACTGCTTCAAAGC | GGCAAATCTCTCGCTGATATTCTTCCAGTTGGC |
| *fat-7* | tttccaccacacattcccac | tcttcacttccgtgattggc |
| *fmo-1* | AAATGATTGGAGCCGACTTG | TCCATTTATGTGGGCCTTTC |
| *ech-9* | ATTCTCGGTTTTGGTTGGCC | ACATCAGTTTGTTGTTCGCAG |
| *gst-29* | CATTTGGCCAAGTTCCAGTT | ATCCGATTTTCCAGCCTTTT |
| K05B2.4 | CCCTATACGAATGACAGGATTG | TGTTTGAACCTTGTGGTGAG |
| *fmo-2* | ggaacaagcgtgttgctgt | gccatagagaagaccatgtcg |
| *ttr-37* | CAGGTGACGACAGAGACGA | TTCAGGGCTGGCTCAATTAC |
| *mdt-15* | CACGACCCGGTCTTTCGTC | CTAGACCACCGCTTGTCTGG |
| *ama-1* | CCTACGATGTATCGAGGCAAA | CCTCCCTCCGGTGTAATAATG |
| *tba-1* | GTACACTCCACTGATCTCTGCTGACAAG | CTCTGTACAAGAGGCAAACAGCCATG |
| *cdc-42* | CTGCTGGACAGGAAGATTACG | CTCGGACATTCTCGAATGAAG |
| *act-1* | GCTGGACGTGATCTTACTGATTACC | GTAGCAGAGCTTCTCCTTGATGTC |
| *ubc-2* | AGGGAGGTGTCTTCTTCCTCAC | CGGATTTGGATCACAGAGCAGC |
| *skn-1* | TCAGGACGTCAACAGCAGAC | GCGAGAGCACATTGATGAC |
| *daf-16* | aacgttccatcatctttccg | gttgcatcgatacgcatttg |

**Table S9: List of worm strains**

| Strain | Reference |
| --- | --- |
| *nhr-49(nr2041) I* | Van Gilst et al. 2005 |
| *nhr-64(ok1957) I* | Liang et al. 2010 |
| *wdr-23(tm1817) I* | Choe et al. 2009 |
| *mdt-15(tm2182) III* | Taubert et al. 2008 |
| *daf-2(e1368) III* | Kenyon et al. 1993 |
| *daf-2(e1370)* *III* | Kenyon et al. 1993 |
| *fat-6(tm331) IV; fat-7(wa36) V* | Brock et al. 2007 |
| LD1171 (ldIs3 [*gcs-1p::gfp* + (*rol-6(su1006)*)]) | Wang et al. 2010 |
| CL2166 (dvIs19[pAF15(*gst-4::gfp::nls*)]) | Leiers et al. 2003 |
| TJ356 (Is DAF-16::GFP) | Henderson & Johnson 2005 |
| CF1553 (muIs84 [(pAD76) sod-3p::gfp + rol-6(su1006)]) | Libina et al. 2003 |
| CF1580 (*daf-2(e1370)* III; muIs84 [(pAD76) *sod-3p::gfp* + *rol-6(su1006)*]) | Libina et al. 2003 |
